# The evolutionary legacy of size-selective harvesting extends from genes to populations

**DOI:** 10.1111/eva.12268

**Published:** 2015-05-27

**Authors:** Silva Uusi-Heikkilä, Andrew R Whiteley, Anna Kuparinen, Shuichi Matsumura, Paul A Venturelli, Christian Wolter, Jon Slate, Craig R Primmer, Thomas Meinelt, Shaun S Killen, David Bierbach, Giovanni Polverino, Arne Ludwig, Robert Arlinghaus

**Affiliations:** 1 Department of Biology and Ecology of Fishes, Leibniz-Institute of Freshwater Ecology and Inland FisheriesBerlin, Germany; 2 Division of Genetics and Physiology, Department of Biology, University of TurkuTurku, Finland; 3 Department of Environmental Conservation, University of MassachusettsAmherst, MA, USA; 4 Department of Environmental Sciences, University of HelsinkiHelsinki, Finland; 5 Faculty of Applied Biological Sciences, Gifu UniversityGifu, Japan; 6 Department of Fisheries, Wildlife, and Conservation Biology, University of MinnesotaSt Paul, MN, USA; 7 Department of Animal and Plant Sciences, University of Sheffield, Western BankSheffield, UK; 8 Department of Ecophysiology and Aquaculture, Leibniz-Institute of Freshwater Ecology and Inland FisheriesBerlin, Germany; 9 Institute of Biodiversity, Animal Health and Comparative Medicine, College of Medical, Veterinary & Life Sciences, University of GlasgowGlasgow, UK; 10 Department of Evolutionary Genetics, Leibniz-Institute for Zoo and Wildlife ResearchBerlin, Germany; 11 Chair of Integrative Fisheries Management, Faculty of Life Sciences, Albrecht-Daniel-Thaer Institute of Agricultural and Horticultural Sciences, Humboldt-Universität zu BerlinBerlin, Germany

**Keywords:** conservation, fisheries-induced evolution, life-history evolution, personality, population dynamics

## Abstract

Size-selective harvesting is assumed to alter life histories of exploited fish populations, thereby negatively affecting population productivity, recovery, and yield. However, demonstrating that fisheries-induced phenotypic changes in the wild are at least partly genetically determined has proved notoriously difficult. Moreover, the population-level consequences of fisheries-induced evolution are still being controversially discussed. Using an experimental approach, we found that five generations of size-selective harvesting altered the life histories and behavior, but not the metabolic rate, of wild-origin zebrafish (*Danio rerio*). Fish adapted to high positively size selective fishing pressure invested more in reproduction, reached a smaller adult body size, and were less explorative and bold. Phenotypic changes seemed subtle but were accompanied by genetic changes in functional loci. Thus, our results provided unambiguous evidence for rapid, harvest-induced phenotypic and evolutionary change when harvesting is intensive and size selective. According to a life-history model, the observed life-history changes elevated population growth rate in harvested conditions, but slowed population recovery under a simulated moratorium. Hence, the evolutionary legacy of size-selective harvesting includes populations that are productive under exploited conditions, but selectively disadvantaged to cope with natural selection pressures that often favor large body size.

## Introduction

Human harvest of wild populations is often intense and nonrandom with respect to phenotypes (e.g. Darimont et al. 2009). In most situations, individuals carrying certain fitness-related traits (e.g. large body size or explorative and bold behavior) are more vulnerable to harvest than others (Allendorf et al. 2008; Alós et al. 2012; Sutter et al. 2012). A well-studied example of human harvest is fishing, which often targets the largest and oldest individuals and is thus positively size selective (Lewin et al. 2006; Jørgensen et al. 2007; Kuparinen and Merilä 2007; Law 2007). Life-history theory suggests that elevated adult mortality favors individuals that allocate energy to reproduction early in life through early maturation at small size and/or increased reproductive investment at the expense of postmaturation somatic growth (Stearns 1992). Such phenotypic changes could be magnified when harvesting is not only intensive but also positively size selective (Laugen et al. 2014). While early maturation increases the probability that an individual will reproduce before it is harvested, small body size at reproduction may confer fitness costs through a decrease in egg number (fecundity), reduced egg and offspring quality (Walsh et al. 2006; Arlinghaus et al. 2010; Uusi-Heikkilä et al. 2010), and increased natural mortality (Jørgensen and Fiksen 2010; Audzijonyte et al. 2013a; Heino et al. 2013; Jørgensen and Holt 2013). However, depending on a species’ ecology and local harvesting patterns, evolution of late, rather than early, maturation (Poos et al. 2011) and fast, rather than slow, growth rate (Walters and Martell 2004; Matsumura et al. 2011; Enberg et al. 2012) can also occur in response to harvesting. Furthermore, if adult mortality is very high and there is thus little fitness to gain by allocating energy to future reproduction, fish might invest heavily in the first reproduction and produce high, rather than low (Walsh et al. 2006), quality eggs and offspring. In fact, despite one might intuitively expect a certain of change in response to size-selective harvesting (e.g. evolution of slower growth rate, Walters and Martell 2004), exact predictions of life-history changes in response to fisheries exploitation are challenging and require stock- and fishery-specific analyses (Arlinghaus et al. 2009; Laugen et al. 2014). Evolutionary changes of body size and related life-history traits can have important repercussions for species and community ecology (Peters 1983; de Roos and Persson 2002; Haugen et al. 2007), management reference points (Heino et al. 2013), and population productivity, recovery speed, and fisheries yield (Law and Grey 1989; Hutchings and Fraser 2008; Conover et al. 2009; Laugen et al. 2014) and thus may be of high relevance to contemporary fisheries management (Conover and Munch 2002; Jørgensen et al. 2007).

A common expectation and empirically reported effect of intensive and size-selective harvesting is the downsizing of body size (Conover and Munch 2002; Jørgensen et al. 2007; Swain et al. 2007; Alós et al. 2014). Beyond correlations between body size and a range of early life-history traits, any selection on body size may also affect underlying physiological and behavioral characteristics through correlated selection responses (Walsh et al. 2006; Uusi-Heikkilä et al. 2008; Diaz Pauli and Heino 2014). Several, not mutually exclusive, mechanisms may be at play. For example, adults may be large because they are efficient in converting energy into somatic growth (an energy conversion mechanism), because they mature late (an energy allocation mechanism) or because they are dominant, bold and aggressive in social interactions and hence superior in securing and defending food resources (an energy acquisition mechanism; Enberg et al. 2012). Large individuals may be also more active and explore the environment more in search for food and they may be able to do so due to lower predation risk (Biro and Post 2008). Any changes in adult body size in response to size-selective fisheries can thus be a consequence of changes in juvenile growth rate (which is an unconfounded measure of growth rate capacity not affected by maturation changes, Enberg et al. 2012), altered maturation schedules (leading to altered energy allocation patterns), or represent an indirect response to selection due to direct selection on correlated behavioral or physiological traits (Walsh et al. 2006; Uusi-Heikkilä et al. 2008; Biro and Stamps 2010; Enberg et al. 2012). Therefore, size-selective fisheries might also induce changes in physiological traits (e.g. metabolism) or behavior (e.g. aggression, boldness) that contribute to energy acquisition and hence growth (Enberg et al. 2012; Sutter et al. 2012; Alós et al. 2015). Most empirical studies on fisheries-induced evolution (FIE) have so far focused on three key life-history traits, namely growth rate, age and size at maturation, and reproductive investment (e.g. Rijnsdorp 1993; Olsen et al. 2004; for reviews, see Policansky 1993; Heino and Godø 2002; Sharpe and Hendry 2009; Devine et al. 2012; Audzijonyte et al. 2013b). Currently, there is little doubt that FIE in the wild could be both plausible and potentially widespread (Jørgensen et al. 2007; Kuparinen and Merilä 2007). However, examination of the joint effects of size-selective fisheries on several traits, including physiology (e.g. metabolism), behavior (e.g. feeding activity) and life history (e.g. growth capacity), in terms of the resulting effects for population dynamics and fisheries has been largely confined to modeling studies (e.g. Thériault et al. 2008; Andersen and Brander 2009; Dunlop et al. 2009; Enberg et al. 2009; Matsumura et al. 2011), and the potential management consequences of FIE remain the least well-understood aspects of FIE (Jørgensen et al. 2007). Notwithstanding the ongoing controversy of whether harvesting causes genetic as opposed to mere phenotypic change, human-induced rapid phenotypic trait change may trigger equally fast ecological change and thereby shape populations, food webs, and ecosystems on a global scale (Darimont et al. 2009; Palkovacs et al. 2012).

Because life-history, morphological, and behavioral traits are at least moderately heritable (Mousseau and Roff 1987), intensive and size-selective fishing over multiple generations is expected to cause genetic (i.e. evolutionary) changes in a range of traits (e.g. Law 2007; Dunlop et al. 2009; Laugen et al. 2014; Marty et al. 2015). Genetic changes, as opposed to mere phenotypic change, may magnify the ecological challenges related to overfishing because they are usually slowly, if at all, reversible in the absence of similarly strong natural selection pressures working in opposite direction than harvest selection (Conover et al. 2009) and thus may have lasting effects on populations and consequently on fisheries. Indeed, meta-analyses, modeling studies, and experimental work have all shown that evolutionary effects of harvesting can impair biomass recovery of overharvested populations (Conover et al. 2009; Enberg et al. 2009; Kuparinen and Hutchings 2012; Neubauer et al. 2013), affect management reference points (Heino et al. 2013), and reduce catchability and hence catch rates and fisheries quality (Philipp et al. 2009; Alós et al. 2015). Fisheries-induced adaptive change might thus have multiple consequences for the population and the fishery, in particular when slowly reversible genetic, as opposed to plastic, changes are involved (Laugen et al. 2014). Despite increasing concern about the effects of size-selective harvesting on wild fish populations (Borrell 2013), the consequences of FIE for populations and fisheries continue to raise controversy (Browman et al. 2008; Jørgensen et al. 2008; Andersen and Brander 2009; see also Bunnefeld and Keane 2014). Although exploited fish populations can consist of individuals of reduced average adult body size for both demographic and evolutionary reasons (Jørgensen et al. 2007), they might remain biologically viable and highly productive precisely because of the evolution of ‘fast’ life histories, that is, early maturation and high reproductive investment (Hutchings 2009; Heino et al. 2013; Jørgensen and Zimmermann 2015). Modeling studies also suggest that if fishing pressure can be kept within optimal limits, FIE is not expected to cause major economic repercussions (Eikeset et al. 2013; Jørgensen and Zimmermann 2015).

The concern about fisheries-induced evolution was first raised at the beginning of the 20th century (Rutter 1902), but has only gained significant momentum since the 1990s when Law and coworkers published their groundbreaking research on FIE (e.g. Law and Grey 1989). Although the potential for rapid FIE is now theoretically, and also empirically, well founded (Jørgensen et al. 2007; Kuparinen and Merilä 2007; Laugen et al. 2014), conclusively detecting it in natural populations has remained a challenge due to the limited opportunities for disentangling plastic and genetic responses in a suite of phenotypic traits (Allendorf et al. 2008; Naish and Hard 2008; Therkildsen et al. 2010; Cuveliers et al. 2011; Pérez-Rodrígues et al. 2013). In theory, experiments on FIE could also be designed in the wild (McAllister and Peterman 1992), but the cause-and-effect mechanism of size-selective harvesting can best be studied experimentally in controlled laboratory environments (Conover and Baumann 2009; Diaz Pauli and Heino 2014). However, to date, only few experimental studies have conclusively reported harvest-induced genetic changes based on quantitative genetics (Conover and Munch 2002; Philipp et al. 2009) or molecular approaches (van Wijk et al. 2013). While there is little doubt that FIE might be occurring in practical fisheries, the magnitude of life-history and other phenotypic changes, the specific genes under selection, and the way how the phenotypic and genetic changes affect fisheries, population viability, productivity, and recovery remain largely unresolved (Jørgensen et al. 2007; Heino et al. 2013; Laugen et al. 2014).

From a conservation and management perspective, fisheries-induced phenotypic changes are of particular concern if they affect population dynamics, viability, and recovery (Hutchings and Fraser 2008; Dunlop et al. 2009; Heino et al. 2013; Laugen et al. 2014). There is ongoing debate whether FIE can significantly affect populations and ecosystem services on timescales that are relevant to fisheries managers (Andersen and Brander 2009; Laugen et al. 2014). Here, we present the results of a selection experiment that provides a comprehensive picture of the evolutionary legacy of size-selective harvesting by examining its phenotypic, genetic, and population-level consequences. A major part of this experiment was to quantify phenotypic and genetic changes in response to five generations of size-selective harvesting in wild zebrafish (*Danio rerio*) in the laboratory using functional genomic markers that can occur close by, within, or in the regulatory areas of genes under selection. Phenotypic changes were scaled up to the population level using a life-history model. Our results provide important insights into FIE, such as how quickly fishing might bring about evolutionary changes, and contribute to the ongoing debate over whether size-selective harvesting causes evolutionary changes in ecologically and economically important phenotypic traits (e.g. in adult body size), and whether these changes matter for biomass renewal, population stability, and conservation.

## Materials and methods

### Size-selective harvesting and breeding design

We used F_1_-generation offspring from approximately 1500 wild-collected zebrafish (parental stock) from West Bengal in India (see Uusi-Heikkilä et al. 2010 for details) in our selection experiment to ensure maximum genetic variation. Our three experimental treatments, each with one replicate (i.e. two tanks per treatment) consisted of approximately 450 zebrafish per replicate tank. We reared individuals in each generation in identical environmental (Supporting Information S1) and density conditions in three replicated selection treatments similar to Conover and Munch's (2002) landmark study. In contrast to Conover and Munch's (2002) design where maturation was triggered by photoperiod and harvesting occurred at a fixed age, the timing of harvesting in our experiment was determined by the maturation schedule of the randomly harvested control treatment: Once 50% of the randomly harvested fish were mature (determined macroscopically from 20 lethally sampled females), we harvested 75% of individuals in all treatments size selectively or randomly according to size. Hence, as not all fish were mature at harvesting, the design represented a harvesting pattern that targeted both mature and immature fish. Prior to harvesting, we measured the standard length (SL) of all fish in all tanks to the nearest mm and wet mass (WM) to the nearest 0.1 g. During harvesting, we sorted the fish by SL and estimated the 75th and the 25th percentiles of the size distributions. To mimic a highly intensive lethal capture fishery (Lewin et al. 2006; Worm et al. 2009; Hilborn and Stokes 2010), we applied a 75% per-generation harvest rate. In the randomly selected line (random with respect to sizes that were harvested), we measured all fish and then assigned the fish randomly with respect to body size to either the harvested group or to the spawning stock. In a large-selected line, we assigned the 25% largest fish to the spawning stocks, and in the small-selected line we assigned the 25% smallest fish to the spawning stocks. The large-selected line hence represented a mortality schedule mimicking a maximum-length limit where the largest mature fish survived. The small-selected line instead represented a positive size-selective harvest scheme common to most fisheries, where young immature fishes are saved because they are not vulnerable to the gear (e.g. too small to become entangled in a fishing net) and/or have to be released due to the minimum-length limit regulations. There was no unharvested control for logistical reasons. A subsample of F_1_-generation fish from each selection line (*N* = 50 per selection line) was measured at age 60 days to ensure that there were no initial differences in body size when the selection experiment was started (large-size selected: 15.2 ± 2.79 mm; randomly selected: 15.0 ± 2.26 mm; small-size selected: 15.8 ± 2.58 mm; mean ± SD; χ^2^ = 1.8105, *P *= 0.4044).

After harvesting, we kept the spawners from each of the six experimental populations in separate aquaria for 14 days to ensure that most fish reached maturity before initiating the spawning trial. To increase the odds of all spawners contributing to the next generation, we mimicked natural spawning conditions (Hutter et al. 2010) by transferring small groups of individuals to spawning boxes. The boxes contained a mesh structure that prevented egg cannibalism (Uusi-Heikkilä et al. 2010, 2012a). We used two sizes of spawning boxes. Five-liter spawning boxes were each stocked with two females and four males, and three-liter spawning boxes were each stocked with one female and two males (altogether 40 females and 80 males per selection line from F_1_- to F_6_-generation). We measured the SL and WM of each spawner before placing it into a spawning box. Females were swapped among boxes once during the spawning trial to ensure a high number of parental combinations and to sustain genetic variation. Spawning trials lasted for 5 days. Each day we cleaned the spawning boxes, placed fertilized eggs on petri dishes, and transferred the petri dishes to an incubator (Tintometer GmbH; *T* = 26°C). We reared hatchlings in the spawning boxes (adults were removed) for 30 days and then transferred them to the rearing tank that their parents were from. The selective harvesting was repeated again when 50% of the randomly selected fish were mature.

We continued the size-selective harvesting for five generations (F_1_–F_6_) and then halted size selection up to four generations (F_7_–F_10_). To estimate the strength of selection during the first five generations, per-generation standard deviation-standardized selection differentials (also known as selection intensity), which describes the difference in the average body size between the spawners and the entire experimental population standardized by the phenotypic standard deviation, were estimated for each generation separately following Matsumura et al. (2012). Note that given our harvesting design, where age at harvest varied from generation to generation in line with potential changes in age at 50% maturation of the random line, did not allow the estimation of selection responses with respect to size at age. From the F_6_-generation onward, 100 individuals per selection line were randomly selected for spawning, and, as in previous generations, their offspring were reared in identical environmental and density conditions. Comparisons of life-history traits among selection lines were conducted two to three generations postselection, and physiological and behavioral traits four generations postselection, in trials where the rearing and growth conditions were strictly standardized among lines. This was performed to remove all confounding maternal and paternal effects and any potential epigenetic effects, thereby increasing the odds that life-history evolution was measured in a comparative way. For example, producing the next generation of fish was time-consuming, and in some cases, populations of our selection treatments were started a couple of weeks apart and consisted of slightly different-aged offspring. Uncontrolled environmental effects during the subsequent holding phase might affect life histories, so the comparison of different traits among the selection lines was performed only after keeping the selected fish for at least two generations without any further selection under controlled and time-matched conditions. Life-history, physiological, and behavioral traits were assessed using offspring from parents that had experienced at least two generations of no selection. For the among-line comparisons, experimental fish of all lines were produced and reared at the same time and in identical conditions. This approach was similar to earlier work in male guppies (*Poecilia reticulata*; van Wijk et al. 2013). Admittedly, delaying the assessment of life-history, physiological, and behavioral traits might not be always desirable in terms of experimental design because the fish might have started converging back to their original life histories as shown by Conover et al. (2009). At the same time, our experimental design can give us some insight into the persistence of potential harvest-induced changes and certainly allowed comparisons among lines that were unconfounded by uncontrolled tank or rearing effects.

### Assessment of life-history, physiological, and behavioral traits

We assessed a range of life-history, physiological, and behavioral traits expected to change in response to size selection after five generations of size-selective harvesting. We focused on life-history traits commonly studied in the context of FIE, namely juvenile and adult growth rates, reproductive investment and maturation schedule (Heino et al. 2013) as well as early life-history traits (Walsh et al. 2006). Moreover, any changes in energy allocation might be related to metabolic changes (e.g. routine metabolism) or changes in energy acquisition patterns related to behavior (exploration and boldness; Enberg et al. 2012). Hence, we also measured standard metabolic rate and measures of risk-taking behavior and exploration in juvenile zebrafish.

#### Growth

To study the growth differences among the selection lines after five generations of size-selective harvesting, we used replicate boxes (each stocked with 10 fish) for each of the experimental populations and their replicate lines (altogether 48 rearing boxes). We measured the SL and wet mass (WM) of the F_9_-generation fish (i.e. third generation after the selection was halted) every 15 days from age 30 days to age 210 days. To derive growth and other growth-related life-history traits, we fitted a biphasic growth model (Lester et al. 2004) to length-at-age data (sexes combined) from each experimental population. The biphasic growth model produced estimates for several key life-history traits, such as juvenile growth rate (*h*), reproductive investment (*g*), age at maturity (*T*), length at maturity (*L*), and asymptotic length (*L*_*∞*_), and expected instantaneous mortality rate (*M*). For more information about the growth experiment and the growth model, see Supporting Information S2.

#### Maturation

To estimate the plasticity in age and size at maturation that stemmed from growth variation among the selection lines, we conducted a maturation experiment in which F_8_-generation fish (i.e. second generation after the selection was halted) were reared under three different feeding conditions (1%, 2%, and 4% of body weight in dry food daily) following the protocol in Uusi-Heikkilä et al. (2011). We used the demographic estimation method (Barot et al. 2004) for estimation of the probabilistic maturation reaction norms (PMRNs; Dieckmann and Heino 2007) for each selection treatment (see Supporting Information S3).

#### Reproductive performance and early life-history traits

We estimated the reproductive performance and potential differences in early life-history traits among the selection lines using F_9_-generation fish. Variables of interest included spawning frequency, clutch size (number of fertilized eggs produced by female, that is, absolute fecundity) and the relative fecundity (number of fertilized eggs per gram of female WM; these three were measures of reproductive performance), egg size, egg survival, larval hatching probability, larval age at hatch, larval length at hatch, larval yolk sac volume, swim bladder inflation probability, and larval survival (these were measures of early life-history traits; Supporting Information S4). In all statistical analyses, selection line was treated as a predictive variable, and selection line replicate, spawning day and couple (i.e. the spawning female and male) were treated as random variables. If there was virtually no variance associated with the random variables, they were excluded from the model. We modeled count data with a Poisson and probability data (e.g. hatching probability) with a binomial error structure. To generate predictions of a potential selection line-specific trait divergence and to control for the effect of female body size on early life-history traits (i.e. size-dependent maternal effects, Hixon et al. 2014), female size was added as predictive variable in a second set of models (except in the larval survival probability where larvae from different spawning couples were pooled). Our approach of running models with and without female size as covariate allowed us to examine whether differences in reproductive traits were associated with maternal body size or whether the traits had evolved independently of maternal size.

#### Metabolic rate

We used juvenile zebrafish of the F_10_-generation (i.e. fourth generation after the selection was halted) to study differences in mass-specific standard metabolic rate (SMR) among the selection lines. Juveniles were used to achieve a measure of base metabolism unaffected by maturation. SMR was measured as rates of oxygen uptake calculated according to a previously published protocol (Dupont-Prinet et al. 2010). We ensured that assumptions of homogeneity and normality of residuals were met, and examined differences in SMR among treatments using a linear mixed model, with selection line as a predictive variable, and selection line replicate as a random variable (Supporting Information S5).

#### Behavior

To study differences in boldness and exploratory behavior as measures of energy acquisition-related behaviors (Enberg et al. 2012) among the selection lines, we used the same fish that were tested for SMR. Each individual was tested twice for its exploration behavior in an open-field test in a novel environment similar to Ariyomo and Watt (2012). In each trial, a single focal fish was introduced into a transparent plastic cylinder in the center of the arena (Supporting Information S5). After a brief acclimatization period, the cylinder was carefully removed and the fish movement was videotaped for 5 min (1st trial). Measurements were repeated for each individual after a break of 30 min (2nd trial). Test fish were measured for total length (TL) and WM after the tests were completed to not stress the fish. As a first proxy for exploration, we calculated the individual mean velocity during each trial (distance moved in 250 s). We further scored the time that a fish spent freezing (defined as not moving faster than 20 mm^−s^) as another proxy for its exploration and degree of boldness (assuming that fish that freeze less are more explorative and bold).

To analyze the data, we first searched for correlations between both response variables (i.e. velocity and time spent freezing) using a principle component analysis (PCA) for both test trials (Supporting Information S5). We used the first principal component (PC1) as a response variable in a linear mixed model in which selection treatment, experimental trial, TL, and WM were predictive variables and individual and selection line replicate were random variables. Model fitting was performed by first evaluating the random effects through likelihood ratio tests. We then excluded all covariates with *P *> 0.1 and refitted the model. In the final model, all other explanatory variables could be excluded, except the selection treatment (predictive variable) and the individual (random variable). More details are given in the Supporting Information S5.

### Evolutionary rate

We estimated evolutionary rates in body size at age 90 days for each selection line using haldanes (Haldane 1949). Haldanes were calculated as: 

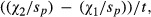
 where *χ* is the mean body length at age 90 days after one generation of selection (F_2_-generation, *χ*_1_) and after five generations of selection and three generation of no selection (F_8_-generation, *χ*_2_), *s*_*p*_ is the pooled standard deviation of trait values across time, and *t* is the number of generations. Mean body lengths at age 90 days were measured from a subsample of fish collected at F_2_- and F_8_-generations.

### Genetic analyses

To determine whether size-selective harvesting induced genetic changes in the experimentally exploited zebrafish populations, we used 371 genomewide, evenly distributed single nucleotide polymorphisms (SNPs) that were chosen from a previously analyzed wild zebrafish dataset (Whiteley et al. 2011) in 502 individuals (Supporting Information S6). Outlier analysis was conducted for F_6_-generation individuals (i.e. first generation after the selection was halted) using the Fdist method (Beaumont and Nichols 1996) implemented in software LOSITAN – Selection Workbench (Antao et al. 2008). We studied the differences in allele frequencies of outlier loci among selection lines with PCA. Because outliers detected by the outlier test can be caused by allele frequency divergence in any number of experimental replicates, we directly examined allele frequency variation for the outlier loci to further characterize the nature of the parallel adaptive divergence. This was performed by creating 95% confidence intervals for the allele frequencies of the selected lines by bootstrapping the allele frequency data. We then determined whether the confidence intervals of the selection lines and selection line replicates overlapped. By doing so for each outlier locus, we identified the selection treatment responsible for the allele frequency differences (and the detection of the outlier loci). We identified genes that were nearby outlier loci on the same linkage group using a SNP database (www.ncbi.nlm.nih.gov) and the Zebrafish Model Organism Database (www.zfin.org).

### Population growth model

We estimated the finite rate of population growth (*λ*) of each selection line under different harvest scenarios (with and without size-dependent harvesting) via a density-dependent Leslie matrix model that incorporated evolved life-history traits (i.e. *h*, *g*, *T*, and *M*, see section ‘*Growth*’ above), fecundity estimates that were based on empirical zebrafish egg weight data, and age-dependent survival probabilities that were determined, with certain adjustments, using empirical estimates of early life-history traits, that is, fertilization rate, egg survival, hatching probability, and larval survival (Supporting Information S7). For simplicity, we modeled only females. We studied the performance of individuals from the large- and the small-selected treatments by comparing the population growth rate of a variant individual in an equilibrium population represented by the randomly selected life history. We introduced three prototypical size-dependent harvest mortality scenarios in the population dynamical model: (i) small-size harvested, which mimicked a harvest slot as harvesting only started on maturing fish and large mature fish were saved, (ii) randomly harvested, which represented unselective harvesting with respect to body size, and (iii) large-size harvested, which represented a standard positive size-selective fishery and/or a fishery managed with a minimum landing size or minimum-size limit (i.e. small-selected experimental fish) (Supporting Information S7; Fig. S2). Actual values of maximum daily instantaneous mortalities and length limits were determined to mimic our experimental 75% per-generation harvest rate. Accordingly, the maximum daily instantaneous mortality (*F*_max_) and the lower (*L*_1_) and upper (*L*_2_) limits of the harvesting scheme for each mortality scenario were as follows: small-size harvested: *F*_max_ = 0.01825 per day, *L*_1_ = 20.8 mm, and *L*_2_ = 24.1 mm; randomly harvested: *F*_max_ = 0.009125 per day, *L*_1_ = 20.8 mm, and *L*_2_ = ∞; and large-size harvested: *F*_max_ = 0.01825, *L*_1_ = 23.7 mm, and *L*_2_ = ∞ (see Supporting Information S7; equation 8, Fig. S2, and Table S10 for details). To reveal potential costs of evolution, we compared the population recovery between the three selection lines after exposure to fishing. To that end, during the first 4000 days (about 30 generations), we introduced size-selective fishing mortality of a similar selectivity as in the experiment (represented by the three harvesting schemes mentioned above) and then stopped fishing and allowed the populations to recover up to 8000 days (about 60 generations). For detailed methodological information of the population growth model, including equations, see Supporting Information S7.

## Results

### Selection intensities

**Figure 1:**
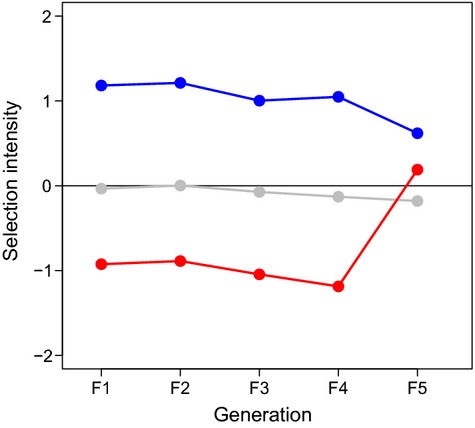
Standard deviation-standardized selection differential (*S*; also known as the selection intensity) estimated for each generation separately. Red, gray, and blue symbols and lines represent populations of small, random, and large fish, respectively.

As expected, we found the harvesting mortality that mimicked positive size-selective fisheries mortality (i.e. small-selected fish) exerted negative standard deviation-standardized selection differentials (i.e. selection intensity) on body length, while the random fish experienced selection intensity close to zero (Fig.1). By contrast, selection for large body size (large-selected fish) exerted a consistently positive standardized selection differential on body length (Fig.1). In the last generation of the selection experiment (F_5_-generation), the selection intensity on body size in the small-selected fish was also close to zero. The reason was that in the last selected generation, the average body size of the small-selected fish in the experimental population was too small (18.7 ± 3.21 mm) for a timely reproduction after selection. To not risk the experiment, it was necessary to apply a slightly higher size threshold (20 mm) when selecting the spawners of the small-selected line. Thus, the small-selected fish only experienced four rather than five generations of intensive size-selective harvest.

### Life-history changes

**Figure 2:**
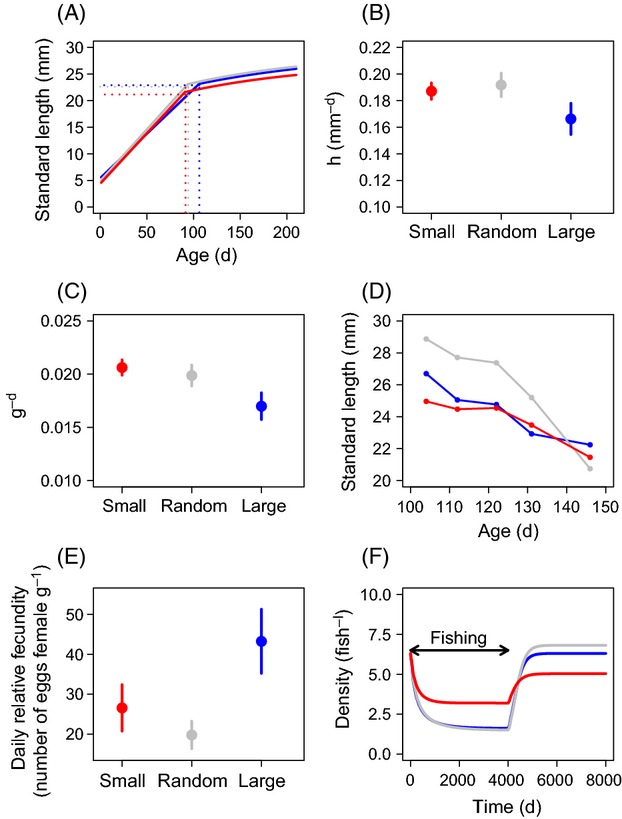
Differences among selection lines in life-history traits and reproductive output. Data are mean ± SEM for panels (B), (C), and (E). Red, gray, and blue symbols and lines represent populations of small-, randomly, and large-selected fish, respectively. (A) Biphasic growth curves (dotted lines refer to treatment-specific age *T* and length at maturity *L*). (B) Average juvenile growth rate (*h*) (small-selected 0.190 ± 0.045; random 0.194 ± 0.040; large-selected 0.167 ± 0.025 mm^−d^). (C) Average daily reproductive investment (*g*) (small-selected 0.021 ± 0.003; random 0.020 ± 0.004; large-selected 0.017 ± 0.005). (D) 50% quantiles for the probabilistic maturation reaction norms (PMRNs; note that most plastic responses in maturation shift the phenotype along the reaction norm, whereas an evolutionary response in maturation shifts the reaction norm itself). (E) Average relative fecundity (small-selected 26.6 ± 5.83; random 19.8 ± 3.44; large-selected 43.3 ± 8.03 eggs/g female). (F) Simulated population-level consequences of life-history changes induced by size-selective harvesting. Population recovery was monitored after large-size harvested fishing was operated for the first 4000 days.

After five generations of size selection followed by three generations of no selection, the small-selected fish had evolved a significantly lower asymptotic length (*L*_*∞*_) (27.4 ± 0.40 mm) compared to the other selection lines (random 29.2 ± 0.29 mm; large-selected 29.5 ± 0.59 mm; Fig.2A, Table1). The small-selected fish were also significantly smaller (9.7 ± 1.96 mm) when the growth experiment started (at age 30 days) compared to random (10.4 ± 2.09 mm) and large-selected fish (10.5 ± 1.87 mm; *χ*^2^ = 15.20; df* *= 4,6; *P *= 0.0005; Fig.2A). Despite the lower maximum length, small-selected (and random) fish exhibited a somewhat, yet statistically not significantly, higher juvenile growth rate (*h*; Fig.2B; Table1). Small-selected and random fish also invested more energy in reproduction (*g*) (Fig.2C, Table1) and matured earlier (*T*) than large-selected fish in the growth experiment (Fig.2A, Table1). Despite the similar age at maturation, small-selected fish matured at a smaller size (*L*) than random and large-selected fish (Fig.2A, Table1). The instantaneous natural mortality (*M*) estimated from the parameters of the biphasic growth model (Supporting Information S2) was higher among small-selected and random (0.018 and 0.017/day, respectively) than among large-selected fish (0.015/day).

**Table 1:** Differences in life-history parameters among the selection lines as estimated by the biphasic growth model

Trait	Selection line	Parameter estimates (SE)	Chi-square value* (df)	*P*-value
Juvenile growth rate (*h*; mm)	Large	0.167 (0.013)	4.8999 (3,5)	0.0863
	Random	0.194 (0.012)		
	Small	0.189 (0.013)		
Maximum length (*L*_*∞*_; mm)	Large	29.53 (0.592)	29.946 (3,4)	<0.0001
	Random	29.24 (0.371)		
	**Small**	27.37 (0.398)		
Age at maturity (*T*; d)	**Large**	106.2 (3.001)	12.490 (3,4)	<0.0001
	Random	94.14 (4.027)		
	Small	91.27 (4.315)		
Length at maturity (*L*; mm)	Large	22.89 (0.449)	0.9742 (3,4)	<0.0001
	Random	22.61 (0.300)		
	**Small**	21.15 (0.322)		
Daily reproductive investment (*g*)	**Large**	0.017 (0.001)	6.4809 (3,4)	0.0109
	Random	0.020 (0.001)		
	Small	0.021 (0.002)		

Parameter estimates for each treatment for each trait are shown. *P*-values were derived from chi-square statistics. The selection line differing significantly from the other treatments is indicated in bold.

* Chi-square value from the deletion of the variable from the full model.

The probabilistic maturation reaction norm (PMRN) describes the 50% probability of maturation as a function of age and size (and potentially other traits affecting maturation) while controlling for the effect of growth on maturation. The maturity ogive used in the estimation of the age-, length-, and condition-based PMRNs included the main effects of age, length, and condition. Condition was a significant factor determining maturity among small- and randomly selected but not among large-selected fish (Supporting Information S3; Table S1). None of the interaction terms in the ogive models were significant. The three-dimensional PMRNs estimated for large- and small-selected fish largely overlapped on the right-hand side of the curve (low growth rate) but not on the left (high growth rate) where small-selected fish exhibited somewhat reduced age and size at maturation (Fig.2D). The PMRN intercept estimated for the random fish was higher than the ones estimated for the small- and large-selected fish, particularly on the left-hand side of the curve, indicating that under fast growth random fish matured at older age and larger compared to the small- and large-selected fish. Variation (measured in standard deviation) in body size across all ages in restricted growth conditions was higher among random fish (SD 3.4 mm; range of SL 9–25 mm) compared to the large-selected (SD 2.7 mm; range of SL 9.0–23 mm) and small-selected fish (SD 2.6 mm; range of SL 8.0–23 mm).

### Changes in reproductive performance and early life-history traits

Without controlling for female body size, small-selected fish of the F_9_-generation had a significantly lower spawning probability (0.28 ± 0.05; mean ± S.E.) than large- (0.51 ± 0.05) and randomly selected fish (0.44 ± 0.05; Table2). Furthermore, small-selected fish produced significantly fewer eggs than random and large-selected fish (lower absolute fecundity), also relative to body size (i.e. relative fecundity, Fig.2E), and overall smaller eggs than large-selected fish (Table2). In terms of relative fecundity, small-selected fish exhibited higher values compared to random fish, but these differences were not significant. Large-selected fish produced slightly larger larvae (3.45 ± 0.013 mm) than small- (3.38 ± 0.021 mm) and randomly selected fish (3.41 ± 0.023 mm), but neither these differences were statistically significant (Table2). Unexpectedly, the offspring produced by random fish had lower hatching probability (0.58 ± 0.03) than offspring of either large- (0.85 ± 0.02) or small-selected fish (0.80 ± 0.03; Table2). The offspring of random fish also took longer to hatch (5.2 ± 0.08 days) than the offspring of large- (4.9 ± 0.05 days) and small-selected fish (4.7 ± 0.08 days; Table2). Other early life-history traits, in particular the larval traits, (Table2) did not differ significantly among the selection lines.

**Table 2:** The effect of the selection treatment on zebrafish reproductive performance and early life-history traits

Trait	Selection line	Parameter estimates* (SE)	Chi-square value† (df)	*P*-value‡
Spawning probability	Large	0.4911 (0.2764)	12.138 (3,4)	0.0005
	Random	0.4437 (0.3160)		
	**Small**	0.2614 (0.3086)		
Fecundity (eggs/female/day)	**Large**	9.1831 (0.4543)	779.16 (3,5)	<0.0001
	**Random**	5.3896 (0.0396)		
	**Small**	3.6352 (0.0354)		
Relative fecundity (eggs/g of female WM/day)	**Large**	14.050 (0.4698)	498.52 (3,4)	<0.0001
	Random	8.5289 (0.0315)		
	Small	8.6313 (0.0246)		
Egg yolk size (mm)	**Large**	0.7286 (0.0116)	82.959 (3,4)	<0.0001
	Random	0.6611 (0.0077)		
	Small	0.6618 (0.0076)		
Egg survival probability	Large	0.9490 (0.5148)	0.958 (3,5)	0.6194
	Random	0.9609 (0.5993)		
	Small	0.9310 (0.5810)		
Hatching probability	Large	0.9037 (0.4083)	8.943 (3,4)	0.0028
	**Random**	0.6177 (0.5684)		
	Small	0.8365 (0.6003)		
Larval age at hatch (days)	Large	4.4977 (0.3788)	8.7742 (5,7)	0.0124
	**Random**	4.9623 (0.5411)		
	Small	4.5052 (0.5820)		
Larval size (mm)	Large	3.6893 (0.1457)	3.2586 (4,6)	0.1877
	Random	3.5411 (0.1218)		
	Small	3.4732 (0.1247)		
Yolk sac volume (mm^3^)	Large	0.0234 (0.0052)	2.5359 (5,7)	0.2814
	Random	0.0171 (0.0045)		
	Small	0.0237 (0.0039)		
Swim bladder inflation probability	Large	0.7561 (0.3926)	1.0741 (4,6)	0.5845
	Random	0.7282 (0.3188)		
	Small	0.7999 (0.3573)		
Larval survival probability	Large	0.9094 (0.2916)	3.4567 (3,5)	0.1776
	Random	0.8284 (0.3893)		
	Small	0.8935 (0.4169)		

WM, wet mass.

Parameter estimates values are given for each treatment and for each trait. Selection treatment differing significantly from the other lines indicated in bold.

* Logit-transformed estimated parameters for binomial distributed data and log-transformed estimated parameters for Poisson distributed data.

† Chi-square value from the deletion of the variable from the full model.

‡ *P*-values derived from the chi-square statistics.

When female body size was added as a predictive variable in the analyses, it explained a significant amount of variation in spawning probability, in absolute and relative fecundity (particularly in small-selected fish), in egg size, and in hatching probability (Table3). Across all selection lines, larger females were more likely to produce eggs more frequently and at higher numbers, but the eggs they produced were smaller and suffered from lower hatching probability. In all of these analyses, except spawning probability, selection treatment remained a significant explanatory variable even after controlling for female size, but again there were few significant effects of either selection treatment or female body size on larval traits (Table3).

**Table 3:** The effect of female body size (standard length; SL mm) and selection treatment on zebrafish reproductive performance and early life-history traits

Trait	Variable	Parameter estimates* (SE)	Chi-square value† (df)	*P*-value‡
Spawning probability	Treatment	0.0047 (0.0454)	0.0249 (3,5)	0.9876
	**Female SL**		18.693 (2,3)	<0.0001
Fecundity (eggs/female per day)	**Treatment**		197.92 (4,6)	<0.0001
	Large-selected	0.0076 (0.5096)		
	Random	0.0049 (0.0404)		
	Small-selected	0.0110 (0.0065)		
	**Female SL**	0.2448 (0.0107)	571.57 (5,6)	<0.0001
Relative fecundity (eggs per g of female WM per day)	**Treatment**		398.15 (4,6)	<0.0001
	Large-selected	0.0748 (0.4892)		
	Random	0.0488 (0.0318)		
	Small-selected	0.1204 (0.0461)		
	**Female SL**	0.1805 (0.0075)	607.74 (5,6)	<0.0001
Egg yolk size (mm)	**Treatment**		21.691 (5,6)	<0.0001
	Large-selected	0.7362 (0.0430)		
	Random	0.7110 (0.0073)		
	Small-selected	0.6793 (0.0087)		
	**Female SL**	−0.0048 (0.0014)	11.817 (6,7)	0.0006
Egg survival probability	Treatment		0.9581 (3,5)	0.6194
	Female SL		0.0011 (5,6)	0.9740
Hatching probability	**Treatment**		12.984 (5,7)	0.0015
	Large-selected	0.9999 (3.4546)		
	Random	0.9998 (0.5575)		
	Small-selected	0.9997 (0.8234)		
	**Female SL**	−0.2796 (0.1146)	5.9779 (6,7)	0.0145
Larval age at hatch (d)	**Treatment**		8.7742 (5,7)	0.0124
	Large-selected	4.4977 (0.3788)		
	Random	4.9623 (0.5411)		
	Small-selected	4.5052 (0.5820)		
	Female SL		1.7207 (7,8)	0.1896
Larval size (mm)	Treatment		3.3456 (4,6)	0.9585
	Female SL		0.0847 (6,8)	0.1877
Yolk sac volume (mm^3^)	Treatment		2.5359 (5,7)	0.2814
	Female SL		1.3175 (7,8)	0.2510
Swim bladder inflation probability	Treatment		1.0741 (4,6)	0.5845
	Female SL		0.1670 (6,7)	0.6828

Parameter estimates are given for the significant covariates, which are indicated in bold.

* Logit-transformed estimated parameters for binomial distributed data and log-transformed estimated parameters for Poisson distributed data.

† Chi-square value from the deletion of the variable from the full model.

‡ *P*-values derived from the chi-square statistics.

### Changes in metabolic rate

Standard metabolic rate (SMR) did not differ among the selection lines (linear mixed model, *F*_2,119_ = 0.157, *P* = 0.855). The average SMR of a standardized fish weighing 0.1 g was 0.0738 ± 0.0053 mg h^−1^ for small-selected, 0.0781 ± 0.0057 mg h^−1^ for random, and 0.0753 ± 0.0053 mg h^−1^ for large-selected fish.

### Changes in behavior

In the behavioral analysis, the PC1 scores for the behavioral traits differed significantly among the selection lines (Supporting Information S5; Table S3). The PC1 captured behaviors (swimming velocity and time spent freezing) that were suggestive of risk taking and boldness. Large-selected zebrafish were significantly more explorative and bolder (i.e. swam with higher mean velocities and spent less time freezing) than small- (*P *= 0.047) and randomly selected fish (*P *= 0.01). Based on the high repeatability value (0.47) for the PC1 score, individuals were highly consistent in their boldness behavior between the 1st and the 2nd trial (χ^2^ = 16.54, df = 1, *P *< 0.001). The consistency may be an indicator of personality.

### Evolutionary rate

The evolutionary rate in body size at age 90 days, estimated as haldanes, was 0.165 for randomly selected fish, 0.053 for the large-selected fish, and −0.116 for small-selected fish.

### Genetic changes

**Figure 3:**
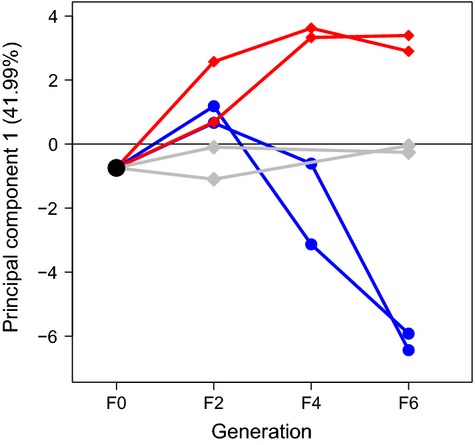
A principal component analysis on allele frequencies of the 22 outlier loci illustrates a similar amount and direction of genetic change occurred among the selection lines induced by size selection after five generations of harvesting. Red lines and symbols indicate small-, gray randomly, and blue large-selected replicates.

Among the 371 SNPs, we identified 22 outlier loci that responded to divergent selection as indicated by high genetic differentiation (*P *< 0.025) (Supporting Information; Table S4). There was also evidence of balancing selection at 12 loci (Supporting Information; Table S5). However, loci under divergent selection are of greatest relevance to studies such as ours and were thus explored in more detail. A PCA on the outlier SNPs found substantial evidence for similar amount and direction of genetic change within each size-selected replicate and relative to the random replicates after five generations of size-selective harvesting (Fig.3).

**Figure 4:**
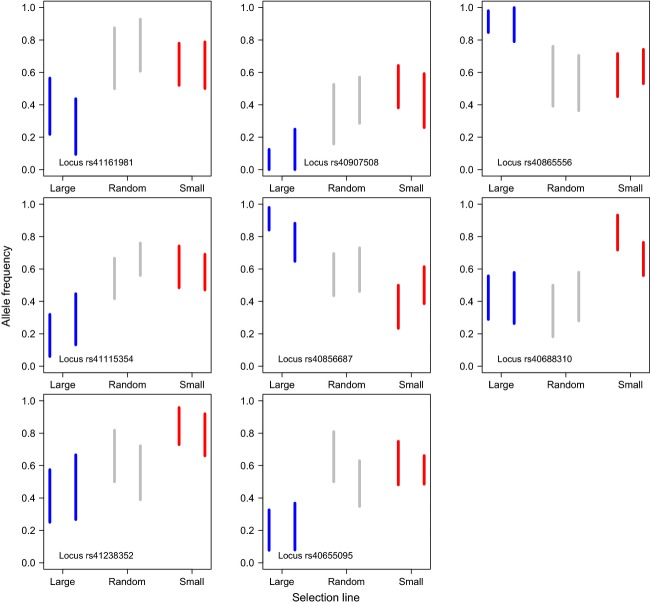
Replicable genetic changes in differently selected zebrafish lines induced by size-selective harvesting indicated by nonoverlapping 95% confidence intervals estimated for allele frequencies for each outlier locus. Large-selected fish indicated with blue lines, random fish with gray lines, and small-selected fish with red lines.

In eight of the 22 outliers, differences in allele frequencies were significant and consistent between the selection line replicates (Supporting Information; Table S6) as there was virtually no overlap in 95% confidence intervals for both size-selected replicates of one selection line (e.g. large-selected) relative to other treatment replicates (e.g. small-selected and random; Supporting Information; Table S6, Fig.4). Parallel allele frequency divergence at these eight loci (hereafter parallel outlier loci) made drift an unlikely explanation and revealed that the mechanistic response underlying adaptive divergence was similar for a subset of loci. For an additional six outlier loci, the significant difference in allele frequency occurred in one of the treatment replicates (Supporting Information S1). Selection may be responsible for these single replicate-specific results, but it is more difficult to rule out genetic drift in this case. Six of the eight parallel outlier loci were in significant linkage disequilibrium (LD; *P* < 0.05) with a nearby SNP on the same linkage group (Supporting Information; Table S7).

Five of the eight parallel outlier SNPs occurred in or close to a gene or in a regulatory region of a gene that has a known function, such as serotonin synthesis, ion transport, regulation of transcription, and collagen formation (Table4). Furthermore, six of the parallel outlier SNPs were in significant LD with another SNP that occurred in a gene or in a regulatory region of a gene with a known function, such as embryonic yolk processing, immune response system, and stress response (Table4).

**Table 4:** Outlier loci with the most pronounced, replicable allele frequency divergence among selection treatments and which occur in or close to a gene with known function

SNP name	Treatment	Type	Gene name	Gene function
rs40907508	Large ≠ small	UTR	LysM	The chemical reactions and pathways resulting in the breakdown of macromolecules that form part of a cell wall
rs40688310	Large and random ≠ small	UTR	Tryptophan hydroxylase 2	The chemical reactions and pathways involving aromatic amino acid family. Controls brain serotonin synthesis in human and mice
rs40655095	Large ≠ small	S	eph receptor B2b	The process of introducing a phosphate group on to a protein. Regulates transcription
rs40856687	Large ≠ small	In	atp1b3b	Ion transport
rs41238352	Large ≠ small	In	col5a1	Collagen formation. Collagen strengthens and supports many tissues, such as bones and muscles
*rs41141381*		*UTR*	*Cathepsin L 1 a*	*Involved in embryonic yolk processing*
rs40878095		*NS*	*Interleukin-1 receptor-associated kinase 4*	*Involved in innate immune response system in zebrafish (Stein* et al. 2007*)*
*rs40784742*		*UTR*	*lims1*	*Involved in cardiac contraction and heart beating*
*rs40769175*		*UTR*	*Guanylate cyclase activator 1C*	*Involved in calcium signaling in the eye and in light regulation, circadian rhythms and stress response (Scholten and Koch* 2011*, Wegener* et al. *2011)*
*rs40739415*		*D*	*Ddt*	*Inner ear development*

Also shown are the type of the variant (S, synonymous, UTR, untranslated region, D, downstream gene variant, In, intron variant), gene name, and gene function.

SNPs in LD with an outlier loci occurring within or close to a gene with a known function are in *italics*.

### Population-level consequences of harvesting-induced life-history evolution

The population model revealed that, in the absence of fishing, small-selected zebrafish had lower population growth rates per day than random or large-selected fish (0.18% and 0.20% lower, respectively; Table5). By contrast, when fish in the model were exploited in a similar positively size-selective manner as in our experiment (as would for example be typical in a minimum-length limit scenario, i.e. small-size selection), the population growth rate per day of small-selected fish exceeded that of the random and large-selected fish (by 0.12% and 0.14%, respectively; Table5). When standardizing the population growth rate by generation time of the random fish, these differences revealed that the population growth rate of small-selected fish was 25.5% lower than the one of random fish in the absence of fishing, while it was 40.5% higher than the one of random fish when exploited with a minimum-length limit. Accordingly, during the period when positively size-selective fishing was operating, small-selected fish showed the slowest population decline among the treatments but when fishing was stopped, the speed of recovery by small-selected fish was slower than that of the other two selection lines (Fig.2F). Moreover, small-selected fish did not recover to the pre-exploitation densities when we assumed no potential for life-history evolution during population recovery (Fig.2F). Finally, small-selected fish did not perform well when random harvesting (with respect to body size) or dome-shaped size-selective harvesting (representing large-selection with a harvest slot-length limit) was operating (Table5, Supporting Information S7; Fig. S3). The modeling results of the relative performance of each of the three life histories were robust to parameter uncertainties (Table5). Although the performance of small-selected fish relative to the other selection lines varied with fishing mortality, small-selected fish outperformed the other selection lines as long as positively size-selective fishing was operating with moderate or high intensity (Fig. S4). Another noteworthy finding was that the population dynamics of the large-selected and the random fish were often quite similar when fishing was operating, suggesting that these two life histories were performing functionally equivalent.

**Table 5:** Comparison of the population growth rate among selection lines under different fishing scenarios

Without fishing		With fishing					
		Small-harvested		Random		Large-harvested	
Large	Small	Large	Small	Large	Small	Large	Small
1.00020	0.99823	0.99991	0.99710	0.99985	0.99915	0.99985	1.00125
(1.00026 ± 0.00081)	(0.99860 ± 0.00120)	(1.00040 ± 0.00295)	(0.99691 ± 0.00337)	(1.00021 ± 0.00063)	(0.99917 ± 0.00081)	(1.00039 ± 0.00145)	(1.00076 ± 0.00192)

Values of large-selected and small-selected fish relative to the random fish are shown. Estimated uncertainty bounds are shown in parentheses (mean ± SD).

## Discussion

Our experimental approach in zebrafish demonstrated changes in genotypes, phenotypes, and population dynamics in response to just five generations of size-selective harvesting. Thus, our results present a comprehensive picture of the evolutionary legacy of size-selective exploitation. Controlled laboratory environment and a specific harvesting design allowed controlling for size-dependent and other parental and epigenetic effects. This helped us to establish unambiguous cause (size-selective harvesting)-and-effect (phenotypic and genetic changes) relationships (Diaz Pauli and Heino 2014) reinforcing the possibility that intensive harvesting of wild populations can indeed lead to fisheries-induced evolution (FIE). Despite introducing obvious simplifications by maintaining discrete generations and allowing only single reproductive events, our selection experiment has value because it allowed the assessment of various phenotypic traits ranging from life-history traits to physiology and behavior and applying a genomic approach to discover specific genes under selection. Five generations of size-selective harvesting of wild zebrafish affected life history and behavior by elevating reproductive investment, decreasing mean maximum body size and reproductive output, and reducing boldness. The evolved phenotypic changes in the small-selected fish were overall relatively subtle and often statistically nonsignificant in relation to the random fish, but they were accompanied by genetic changes and large population dynamical effects. These results collectively showed that contemporary harvest-induced evolution is conceivable in response to intensive size-selective exploitation. The population model further revealed that the phenotypic and genetic changes induced by positive size selection allowed fish to adapt to harvesting, but hindered population recovery in the absence of exploitation. Our results overall highlight the potential for large, harvest-induced population-level consequences to emerge from rather subtle phenotypic changes in response to positively size-selective exploitation that might easily go unnoticed when monitoring natural populations.

We examined the outcome of five generations of size-selective harvesting by comparing the phenotypes of individuals among selection treatments several generations after selection was halted. Hence, our results are conservative because delaying the trait assessment up to four generations without harvesting probably had resulted in some recovery of phenotypic traits due to fecundity selection similar to the case in the famous Atlantic silverside (*Menidia menidia*) experiment (Conover et al. 2009; Salinas et al. 2012). Moreover, for logistical reasons, we only exerted negative selection differentials on body size for four rather than five generations in our fishing treatment (the small-selected line, Fig.1), further reducing the potential for phenotypic (and genetic) change. We nevertheless documented effects of positively size-selective harvesting on a range of traits and also presented molecular evidence of directional selection altering the genotypes, reinforcing the previously expressed notion that rapid evolutionary change is possible over very short time periods of intensive size-selective harvesting (Conover and Munch 2002; van Wijk et al. 2013).

### Evolved differences in growth and behavior

According to the biphasic growth model, small-selected fish (under selection similar to most capture fisheries managed with minimum-length limits) reached significantly lower mean maximum body size (*L*_*∞*_) than large-selected and random fish, but there was no significant difference in early growth rate (*h*) among the selection lines (Fig.1A,B). These findings were in line with recent field evidence in heavily exploited coastal marine fish species (Alós et al. 2014) and have been also reported elsewhere (Nússle et al. 2009). Early maturation at small size and high reproductive investment can together explain the lower *L*_*∞*_ of the small-selected fish given the fundamental energetic trade-off between growth and reproduction (Enberg et al. 2012). In addition to such differences in energy allocation, juveniles of the small-selected fish evolved differences in energy acquisition because they were significantly less bold in an open-field experiment compared to large-selected fish. These results suggest that the small-selected zebrafish evolved a more cautious behavioral type and personality, likely in relation to feeding behavior, which might have contributed to their lower body size at harvesting. Similarly, small-selected fish in the Atlantic silverside study evolved lower food consumption and were less willing to forage under threat of predation (Walsh et al. 2006). Variation in exploration and boldness can have fitness consequences because these behaviors can facilitate foraging success (Stamps 2007; Klefoth et al. 2012), dispersal (Cote et al. 2010), cognitive performance (Vital and Martins 2011), reproduction (Ariyomo and Watt 2012), and survival (Smith and Blumstein 2007; Biro and Stamps 2008). Moreover, boldness and exploration relate directly to vulnerability to fishing gear (Alós et al. 2012, 2015; Härkönen et al. 2014; Diaz Pauli et al. 2015). Therefore, increased timidity as an evolutionary response to size-selective fishing will negatively affect catch rates (Philipp et al. 2009), reduce angler satisfaction (Arlinghaus et al. 2014), and potentially also affect the economic value of a fishery. Moreover, when catchability declines so does the value of fishery-dependent information to index stock size (Alós et al. 2015).

A common assumption in the fisheries literature is that size-selective mortality should reduce growth rate (Conover and Munch 2002; but see Walters and Martell 2004 for a critical view of this apparently intuitive prediction). By contrast, we found that small-selected zebrafish did not differ significantly in their juvenile growth rate compared to random and large-selected fish, and there were also no physiological differences in standard metabolic rate (and, therefore, in physiological growth capacity) among the selection lines. In fact, there was a tendency for small-selected and random fish to grow faster than large-selected fish (Fig.1B). This agrees with modeling work (Matsumura et al. 2011) and empirical data in positively size selectively exploited fisheries (van Walraven et al. 2010). The lack of significant differences in juvenile growth (which, unlike the adult growth rate, is a clean measure of growth capacity; Enberg et al. 2012) could have been caused by our harvesting design where each fish could only spawn once. Hence, in the random line, larger females, which also carried more eggs due to the positive relationship of female size and fecundity (hereafter referred to as fecundity selection), after harvesting were likely selectively favored, in turn likely creating a selection pressure on fast juvenile growth rate despite a nonselective harvest pattern relative to size. Similarly, in the small-selected line, the fastest growing fish probably contributed more eggs to the next generation and this maintained positive selection pressure on fast juvenile growth. Thus, harvesting alone, even when nonselective, could have had a similarly strong effect as size-selective harvesting on the evolution of fast juvenile growth through fecundity selection (Engen et al. 2014). In other words, the lack of substantial differences in juvenile growth rate among random and the small lines could be indicative of a lack of additional evolution of size-selective harvest compared to unselective harvesting. Not having a nonharvest control precludes our ability to fully understand the relative effects of selection pressures on juvenile growth rate caused by unselective versus size-selective harvesting. Nevertheless, our results support theoretical arguments and empirical data that one should not generally assume that size-selective harvesting will cause evolution toward low growth rates (Walters and Martell 2004; van Walraven et al. 2010; Enberg et al. 2012). In fact, the opposite can and will occur in many situations because when adult mortality rate is elevated, it is advantageous to be as large as possible on the first spawning attempt to outpace the high mortality rate with higher reproductive output (Boukal et al. 2008; Dunlop et al. 2009; Matsumura et al. 2011), as in our experiment.

The weak response of juvenile growth rate of small-selected fish after five generations of size-selective harvesting is in contrast to a groundbreaking experimental study on harvest-induced selection in Atlantic silversides by Conover and Munch (2002), who reported a steep decline in (juvenile) growth rate after four generations of size-selective harvesting. Although silverside and zebrafish share many life-history and behavioral characteristics (e.g. high fecundity, small egg size, external fertilization, and schooling behavior), silverside are semelparous, while zebrafish are iteroparous batch spawners. These differences in life-history strategies could strongly affect energy allocation patterns and thus juvenile growth. Silverside is a capital-breeding species that uses stored energy to make large investments into reproduction, and females typically reproduce in years when they have accumulated a threshold level of stored energy reserves (Bull and Shine 1979). By contrast, income breeders, such as zebrafish, spend energy on reproduction as it is gained (Jönsson 1997). Furthermore, Conover and Munch (2002) exerted a greater harvesting pressure (90% per generation) compared to the present experiment in zebrafish (75% per generation), and this might have affected the results by increasing the selection response in juvenile growth rate. Most importantly, however, in the silverside study, juvenile traits were exclusively under selection because maturation was induced by photoperiod after the experimental harvesting (Diaz Pauli and Heino 2014). This experimental procedure channelized selection differentials on juvenile growth rate, in contrast to the present case in zebrafish where reproductive traits, in particular reproductive investment, were allowed to be under selection in addition to juvenile growth rate. A recent selection experiment in male guppies (*Poecilia reticulata*) similarly demonstrated evolution in maturation and only a minor change in juvenile growth rate after only three generations of selection (van Wijk et al. 2013). However, that study differed from ours because it focused on determinately (rather than indeterminately) growing males (rather than males and females combined). In general, however, responses of juvenile growth rate to harvesting vary among species and fisheries, and one should not necessarily expect juvenile growth rate to decline in response to positively size-selective harvesting (Walters and Martell 2004; Enberg et al. 2012).

### Maturation

While reproductive investment increased in response to selection for small body size in our experiment, we saw little differentiation in the maturation schedule (represented by the probabilistic maturation reaction norm, PMRN; Heino et al. 2002; Dieckmann and Heino 2007) between small- and large-selected zebrafish after five generation of size-selective harvesting. It is noteworthy, however, that although the PMRNs of small- and large-selected fish largely overlapped, investigation of the left part of the PMRNs, that is, the area where food was abundant and growth rate was high, indicated that small-selected fish matured somewhat earlier and at smaller size than large-selected zebrafish (Fig.2D). This pattern was consistent with the predicted reduced age at maturation (*T*) that we estimated from growth under *ad libitum* food conditions (Table1). The high size- and age-specific maturation probability of random fish could have been affected by unselective harvesting. Despite we lacked a nonharvest control, the evolved differences in the random fish still represent adaptation to unselective fishing. However, we based the timing of harvesting on the 50% maturation status of the random fish; thus, one should have expected a lower PMRN intercept compared to the large-selected fish as indicative of earlier maturation. It is more likely that the higher size- and age-specific maturation probability in the random line was caused by the large-size variation compared to large- and small-selected fish. In zebrafish, social dominance is size dependent (Paull et al. 2010) and in random fish the variation (measured as standard deviation) in body size during the maturation experiment across all ages was substantially higher (3.0 mm), particularly in growth-restricted conditions, compared to small- (2.5 mm) and large-selected fish (2.5 mm). Accordingly, the higher size- and age-specific maturation probability of the random fish could have been caused by size-dependent social hierarchies or other social factors, which have been shown to inhibit and delay reproduction for example in guppies (Diaz Pauli and Heino 2013).

While the evolution of elevated reproductive investment *g* (Fig.2C) and lower *L*_*∞*_ (Table1) in response to size-selective harvesting among small-selected fish was consistent with empirical and theoretical studies (e.g. Rijnsdorp 1993; Jørgensen et al. 2007; Sharpe and Hendry 2009; van Walraven et al. 2010), the lack of substantial difference in the maturation schedule caused by selective harvesting (as inferred from the PMRN) may seem counterintuitive. However, our experiment was based on nonoverlapping generations; hence, each selected spawner could contribute to the next generation just once during one spawning event at a fixed age. Therefore, our experiment prevented early-maturing fish from benefiting from the increase in spawning frequency, which is a key fitness benefit associated with early maturation when adults face a high risk of mortality (Poos et al. 2011). The conditions in our experiment were thus artificial and in contrast with the conditions in the wild. In the wild, fisheries maturation schedules have been found to readily respond to harvest selection as indicated by large changes in the PMRN's intercepts and slopes in many stocks, and these changes were often much more pronounced than changes in other life-history traits, such as reproductive investment (Hutchings and Fraser 2008; Sharpe and Hendry 2009; Devine et al. 2012; Audzijonyte et al. 2013b). Therefore, it is important not to misinterpret our results as evidence that elevated or size-selective mortality does not alter the maturation schedule of exploited fish species. In fact, evolutionary response in maturation is expected under most exploited conditions when generations overlap (Devine et al. 2012). Further experiments with overlapping generations are needed to fully understand how maturation will evolve in response to size-selective harvesting (Diaz Pauli and Heino 2014).

### Evolved differences in reproductive success and early life-history traits

Evolutionary downsizing of adult body size, especially of females, can have large consequences for offspring production and larval viability (Johnson et al. 2011), for example through direct fecundity decline associated with the reductions in adult body size or indirectly through size-dependent maternal effects on egg and offspring quality (Walsh et al. 2006; Arlinghaus et al. 2010; Hixon et al. 2014). In our study, we demonstrated a positive association between maternal body size and reproductive output (spawning probability and fecundity; Table3). Larger females had a higher spawning probability, which was exclusively determined by female body size and not affected by the selection treatment. Female body size was also positively associated with fecundity. Large females of many fish species have been found to have higher amount of energetic resources to allocate to reproduction compared to small females and thus are able to spawn more frequently and produce a higher number of eggs (Hixon et al. 2014), in line with our findings in zebrafish reported here and elsewhere (Uusi-Heikkilä et al. 2010). Maybe somewhat surprisingly egg size was negatively associated with maternal body size. This could represent a fundamental egg number – egg size trade-off and relate to smaller females compensating their lower fecundity by producing larger eggs (Hendry et al. 2001; Uusi-Heikkilä et al. 2010). Similarly, the negative relationship between female size and hatching probability could be related to the above-mentioned trade-off. However, the biological relevance of egg size as a trait of egg quality has been questioned before in zebrafish (Uusi-Heikkilä et al. 2010). Irrespectively, when early life-history or reproductive traits correlate with the focal trait under selection (i.e. adult body size), these traits can respond via correlated selection response due to genetic covariance (Munch et al. 2005). We found significant differences in fecundity (but not in spawning probability) and in several early life-history traits (egg size, hatching probability, and larval age at hatch) among the three selection lines even after statistically controlling for maternal body size, suggesting an evolutionary response unrelated to size-dependent maternal effects.

After just five generations of selection, large-selected fish produced more and larger eggs compared to small-selected and random zebrafish, and there was a modest nonsignificant increase in relative fecundity in small-selected fish compared to random fish (Fig.2E). As reproductive investment increased in the small-selected line compared to the random line, this likely compensated for the evolution of smaller adult body size and maintained fecundity high and largely unaltered with respect to the random line. It is surprising that there were such large differences in fecundity between random and large-selected zebrafish despite their similar maximum length (Fig.2A). These differences could have been caused, at least partly, by size-dependent dominance hierarchies. In zebrafish, dominance and aggression are positively associated with increasing body size (Paull et al. 2010). Larger differences in spawner body sizes in random females (SD 1.99 mm; range 24–32 mm) compared to large-selected females (SD 1.37 mm; range 27–31 mm) could have maintained higher level of aggression between females and males in the spawning boxes occupied with random fish and resulted in lower egg production. Also, males being larger than females, which by chance should have been occurred more often in the random line given the larger size range of the spawners, could have affected egg production due to female stress caused by male dominance (D. Bierbach, S. Uusi-Heikkilä, P. Tscheligi, C. Wolter and R. Arlinghaus, unpublished data). Furthermore, zebrafish females allocate more reproductive resources to more preferred, large males (Uusi-Heikkilä et al. 2012b), and because in large-selected line males were generally larger, large-selected females could have released more eggs toward them compared to random line, where fewer females were coupled with a large male.

Similar to the fecundity assessments, it is also noteworthy that there were no large differences in egg traits between the small-selected and random fish. Earlier studies in zebrafish showed that egg size might not be a major determinant of larval quality (Uusi-Heikkilä et al. 2010). Instead, offspring quality may be better indicated by larval traits such as age at hatch, size at hatch, and the amount of nutrient reserves (yolk sac volume). Large-selected and random fish produced slightly larger larvae than small-selected fish, but the differences were not statistically significant and the differences neither translated into shorter hatching time as an indicator of better larval condition and faster development rate (Kimmel et al. 1995). Hence, size-selective fisheries selection did not substantially alter egg and larval traits when comparing the small-selected fish with the random line. However, random fish had a significantly greater spawning probability than small-selected fish, suggesting that the random fish still might have greater reproductive fitness compared to the small-selected fish.

Despite the lack of differences in early life-history traits and juvenile growth rate, our results suggest that random and large-selected fish exhibited faster larval growth than small-selected fish. This was indicated by the large differences in body at age 30 days (Fig.2A) when the growth experiment started. Despite their similar sizes at hatch (Table2), random and large-selected fish were significantly larger at age 30 days compared to small-selected fish. Fast larval growth has fitness benefits because it allows larvae to pass the most vulnerable life-history stages quickly, and although there might be some costs related to fast growth (e.g. increased larval mortality; Pepin 1991), it has also been suggested that even slightly enhanced rates of early survival and growth can translate into increased probability of subsequent recruitment (Houde 1987; Hare and Cowen 1997).

Overall, the differences in early life-history traits among zebrafish selection lines were substantially smaller (and often nonsignificant) compared to those previously reported in the silverside study (Walsh et al. 2006). The inconsistency between the silverside and our study is probably related to the steeper decline in the body size of mature silversides after four generations of harvesting and to the larger difference in body size between small- and large-selected fish during the spawning trials compared to our zebrafish study. In fact, large-selected silversides were more than six times heavier than small-selected ones, whereas in our study the differences were less than twofold. Moreover, the differences between these two studies could again be related to the different life-history strategies of the two species. Semelparous silversides may invest a much larger proportion of surplus energy into a single reproductive season compared to zebrafish, which spread their reproductive effort over multiple batches. Different results among experimental evolutionary studies reinforce the species and environment specificity of FIE, which complicates the formulation of general predictions about the type and magnitude of phenotypic changes as a result of size-selective harvesting (Heino et al. 2013; Laugen et al. 2014).

### Evolutionary rate

The degree of decline in adult body size of small-selected fish in our experiment (7.8% over five generations of selection) was similar to recent experimental work in male guppies exposed to three generations of size-selective mortality (7% over three generations; van Wijk et al. 2013) but differed substantially from the silverside study (25% over four generations; Conover and Munch 2002). Although the change in adult body size in our study was significant, such a subtle phenotypic change in body length might easily go unnoticed in phenotypic time series from the wild because fish growth has a large plastic component (Lorenzen and Enberg 2002). In addition, the rate of evolution of adult body size that we observed was lower (−0.116 to 0.165 haldanes) than the rate estimated in the male guppy experiment (0.3 haldanes; van Wijk et al. 2013) and much lower than those estimated for size at maturation from actual fisheries data (−2.2 to 0.9 haldanes; Devine et al. 2012). Thus, the phenotypic changes that we observed in the laboratory were conservative relative to the data from the wild, possibly because the latter include both genetic and plastic changes and because selection in overlapping generations may lead to stronger responses in maturation traits than revealed in our experiment. Our finding underscores an important dilemma for FIE studies: While FIE can be widespread in exploited systems (Jørgensen et al. 2007; Devine et al. 2012; Laugen et al. 2014), it is very difficult to detect when one is confined to just phenotypic analysis, given that fish life-history traits are extremely plastic and vary in response to the environment (Kuparinen and Merilä 2007; Law 2007). This issue has potentially contributed to the lack of broad acceptance of FIE among fishers, fisheries managers, and some fisheries scientists (Jørgensen et al. 2007; Kuparinen and Merilä 2007; Law 2007; Hilborn and Minte-Vera 2008).

### Harvest-induced genetic changes

In the genetic analyses, we identified 22 outlier loci responding to divergent selection (Table S6) and 12 outliers indicative of balancing selection (Table S7). The outliers indicative of balancing selection were linked to genes involved in processes such as movement of metal ions within a cell or between cells (Varshney et al. 2013), proteolysis, neuronal development (Ahrens et al. 2012), and glycopeptide hormone activity (Alderman and Bernier 2007). These loci with signatures of balancing selection in F_7_-generation might have responded to laboratory rearing environment (i.e. captivity). Another possibility is that they are associated with fecundity selection likely experienced by all experimental lines. Among the 12 loci that exhibited significant signatures of balancing selection, three can be considered as candidates for a fecundity selection hypothesis (Table S7). None of these loci was directly associated with genes related to reproduction or fecundity (Table S7), although they could be in linkage disequilibrium (LD) with genes that are. Despite the fact that the loci with signatures of balancing selection might have been indicative of adaptation to the laboratory environment, at least some of them might have been false positives. Inaccurate detection of balancing selection is an inherent weakness of outlier approaches (Narum and Hess 2011), and the method employed here, in particular, has been shown to have relatively high type I error rate for balancing selection (Beaumont and Nichols 1996).

Our results provide conclusive evidence that size-selective harvesting can lead to genetic change in timescale relevant to fisheries. We conservatively focused on the eight outliers that showed parallel divergence in both size-selected replicates of one selection line. These outliers and adjacent loci emerged as the strongest candidates of adaptive divergence. Five of the eight parallel SNPs occurred within or close to a gene with a known function (Table4) and six of them were in significant LD with a nearby SNP on the same linkage group (Table4). Two of the eight outliers were in LD with a SNP that occurs within a regulatory area of a gene associated with zebrafish embryological metabolism (Tingaud-Sequeira and Cerdà 2007) and two other outliers with SNPs occurring within regulatory areas of the genes or within genes associated with zebrafish circadian rhythms, stress response, and immune system (Stein et al. 2007; Scholten and Koch 2011; Weger et al. 2011). These traits might be important in determining adaptive responses related to fitness in juvenile and adult fish. Another parallel outlier was located within a regulatory area of a gene, which has been found to control brain serotonin synthesis in humans and mice. Serotonin is a key element in the synthesis of melatonin, a hormone that affects feeding behavior and aggression in fish (Falcón et al. 2010). Differences in melatonin production could relate to differences in fish exploration tendency, which was found to evolve in our experiment. Denser SNP panels and mapping approaches would be needed to test the functional role of these genes along with other genes with which they are in LD.

### Population-level consequences

The consequences of even subtle phenotypic changes for populations could potentially be severe. For example, early maturation and high investment in reproduction cumulatively reduce life span (Jørgensen and Fiksen 2010). Indeed, the estimated instantaneous natural mortality (*M*) of zebrafish was higher among small-selected and random fish than among large-selected fish. According to our population model, under positively size-selective fishing (minimum-length limit scenario), the small-selected fish population would exhibit a substantially greater population growth rate than the random and large-selected fish populations. This finding supports the idea that life-history changes are compensatory in that they allow individuals (and therefore populations) to remain productive in the face of positively size-selective harvest mortality (Hutchings 2009; Matsumura et al. 2011; Kuparinen and Hutchings 2012; Heino et al. 2013). Hence, from a fisheries perspective, FIE is not necessarily negative (Eikeset et al. 2013; Jørgensen and Zimmermann 2015). However, in our model during a simulated fishing moratorium, the small-selected fish population exhibited a substantially lower population growth rate than the random and large-selected fish populations (Fig.2F and Fig. S2). Our modeling results therefore suggest that FIE impedes population recovery during a moratorium, which is in line with previous empirical and theoretical research (Conover et al. 2009; Enberg et al. 2009; Eikeset et al. 2013; Hutchings and Kuparinen 2014; Kuparinen et al. 2014; Laugen et al. 2014; Marty et al. 2015). Thus, seemingly subtle changes in life-history traits could have a strong effect on the recovery rate and rebound potential of exploited fish populations. Moreover, evolutionary downsizing in body size of only 0.1% per year over 50 years has been predicted to reduce biomasses up to 35% in some species (Audzijonyte et al. 2013a). Hence, FIE matters for the management and conservation of exploited fish populations, even if phenotypic responses are modest and seemingly unimportant at an individual level.

Our population model was simplified by design and therefore subject to caveats. Although the model included knowledge of density dependence of vital rates in zebrafish, it used the dominant eigenvalue of the Leslie matrix as a fitness metric although the dominant Lyapunov exponent has been suggested as an appropriate measure in density-dependent population models (Roff 2010). In addition, the model did not incorporate the potential effects of dominance hierarchies and female differential allocation on zebrafish reproductive output (Uusi-Heikkilä et al. 2012b). Moreover, model results were based on the assumption that the parameters for growth, maturation, and reproductive investment that we measured in the laboratory would translate to field conditions. Our approach to fitting the biphasic growth model (Lester et al. 2004) allowed us to predict numerous life-history traits that could be incorporated into the model. However, our approach assumed that these traits were optimally adapted to treatment conditions. Our experimental populations might not have reached an evolutionary stable state after five generations of selection. Nevertheless, results of the among-population comparison of population growth rate should be robust to this omission because the population model itself does not require the assumption of evolutionary equilibrium and the estimated population growth rates were rather insensitive to uncertainty of the parameter values of the Lester growth model (i.e. growth *h*, reproductive investment *g*, and maturation *T*). Finally, we were not able to perform an evolutionary impact assessment (Jørgensen et al. 2007; Laugen et al. 2014) in a strict sense because we lacked preharvest life-history data and were unable to compare population-level effects over time with and without evolution. We thus could not evaluate the full implications of FIE. Nevertheless, there is value in comparing fitness of evolved life-histories (both small- and large-selected fish) relative to the random fish, and we can interpret our population dynamical results as showing the effect of size-selective harvesting relative to unselective harvesting.

## Conclusions and implications

Much of the current debate around the prevalence of FIE has centered on whether the observed phenotypic changes are genetic (Jørgensen et al. 2007; Kuparinen and Merilä 2007; Law 2007) and if so, whether these changes matter for population dynamics and hence management (Hutchings and Fraser 2008; Andersen and Brander 2009; Kuparinen and Hutchings 2012; Laugen et al. 2014; Marty et al. 2015). The strength of our experimental study is that it establishes an unambiguous cause-and-effect relationship by showing that (i) size-selective harvesting can lead to genetic and a range of phenotypic changes in contemporary timescales, (ii) a relatively low evolutionary rate, and (iii) seemingly subtle phenotypic changes in individual life-history traits can cumulatively have a strong effect on population growth rate and recovery potential. FIE can help to maintain a productive population while harvesting is intensive, but our results suggest that the same population adapted to exploitation is expected to recover slowly and may not reach pre-exploitation levels when fishing is relaxed. Our work on the evolutionary legacy of size-selective harvesting thus reinforces the notion that the potential for FIE and its population-level consequences are of relevance to fisheries management and conservation. Negative consequences of FIE will be particularly large for stocks that have been poorly managed in ecological and economic terms (Eikeset et al. 2013; Jørgensen and Zimmermann 2015) for a long period of time (Neubauer et al. 2013), and in such cases, it is critical that the ecological and evolutionary consequences of fishing are being carefully evaluated and mitigated.

A straightforward measure that can help curtail the largely inevitable FIE (Matsumura et al. 2011) is to carefully control fishing mortality to keep it within ecologically sustainable and economically optimal bounds as shown in two recent modeling studies in a FIE context (Eikeset et al. 2013; Jørgensen and Zimmermann 2015). A second complementary measure could be to manage the fishing-induced selectivity, which may produce positive outcomes from a human perspective (e.g. evolution of large adult body size as opposed to downsizing of adults, Boukal et al. 2008; Jørgensen et al. 2009; Matsumura et al. 2011). For example, we found that the population dynamics of large-selected fish, which evolved large asymptotic adult body size, did not differ from the random fish in any of the modeled fishing scenarios (Fig.2F and Fig. S2). Large-selected fish were exposed to a maximum-size harvest; thus, our results could be interpreted that saving large fish selects for life histories that are more similar to unselectively exploited fish compared to a strictly positively size-selective exploitation common with minimum-length limit regulations and in most other real fisheries. Previous modeling studies have also emphasized a superior performance of harvest slots (i.e. dome-shaped selectivity where large fish and small fish are saved from harvesting) over standard minimum-length limits in terms of reducing selection responses in maturation and other traits while facilitating evolution of large adult size under certain conditions (Hutchings 2009; Jørgensen et al. 2009; Matsumura et al. 2011). Therefore, when feasible and desired by stakeholders, the implementation of maximum-size limits or harvest slots at the expense of using minimum-size limits could be recommended as an additional measure of altered selectivity patterns to complement management measures directed at controlling fishing mortality.

## Data archiving statement

Data available from the Dryad Digital Repository: http://dx.doi.org/10.5061/dryad.r4q8j.

## Supporting Information

**Figure S1.** Outlier loci, which showed no replicable (consistent) response to selection but were detected by the outlier test (FDist method).

**Figure S2.** Three typical size-selective fishing scenarios (see Table S10 for the parameter values).

**Figure S3.** Simulated population-level consequences of life-history changes induced by size-selective harvesting.

**Figure S4.** Sensitivity of the population growth rate ʎ.

**Appendix S1** Methods and results. **Table S1.** Final growth and condition models with their covariates and *t*-statistics for each selection line. **Table S2.** Maturity ogive models for each selection line. **Table S3.** Differences in PC1 score (estimated marginal means) derived from the final model among the selection lines. **Table S4.** The SNP name, chromosome location, map position (Mb), *F*_ST_-value, heterozygosity and the adjusted *P*-value (for empirical *F*_ST_ higher than simulated *F*_ST_, false discovery rate set at 0.1, at 95 % confidence interval level) of the outlier loci detected using the FDist method. **Table S5.** Outlier loci responding to balancing selection: type of the variant (UTR = untranslated region, D = downstream gene variant, I = inter-genic variant, In = intron variant), gene name, and gene function. **Table S6.** Outlier loci with the most pronounced, replicable allele frequency divergence among selection treatments. **Table S7.** Outlier SNPs that were in linkage disequilibrium (LD) with a nearby SNP (LD SNP) and the physical distance in kilobase pairs between them. **Table S8.** Maturation- and growth-related parameters estimated for each treatment based on the biphasic growth model (Lester et al. 2004). **Table S9.** Model parameters used in the zebrafish Leslie-matrix model. **Table S10.** Three typical size-selective fishing scenarios.
