# Supplementary figures and images for: The evolutionary legacy of size-selective harvesting extends from genes to populations
Source: Evol Appl. 2015 May 27;8(6):597–620. doi: 10.1111/eva.12268 (PMC4479515; doi:10.1111/eva.12268)

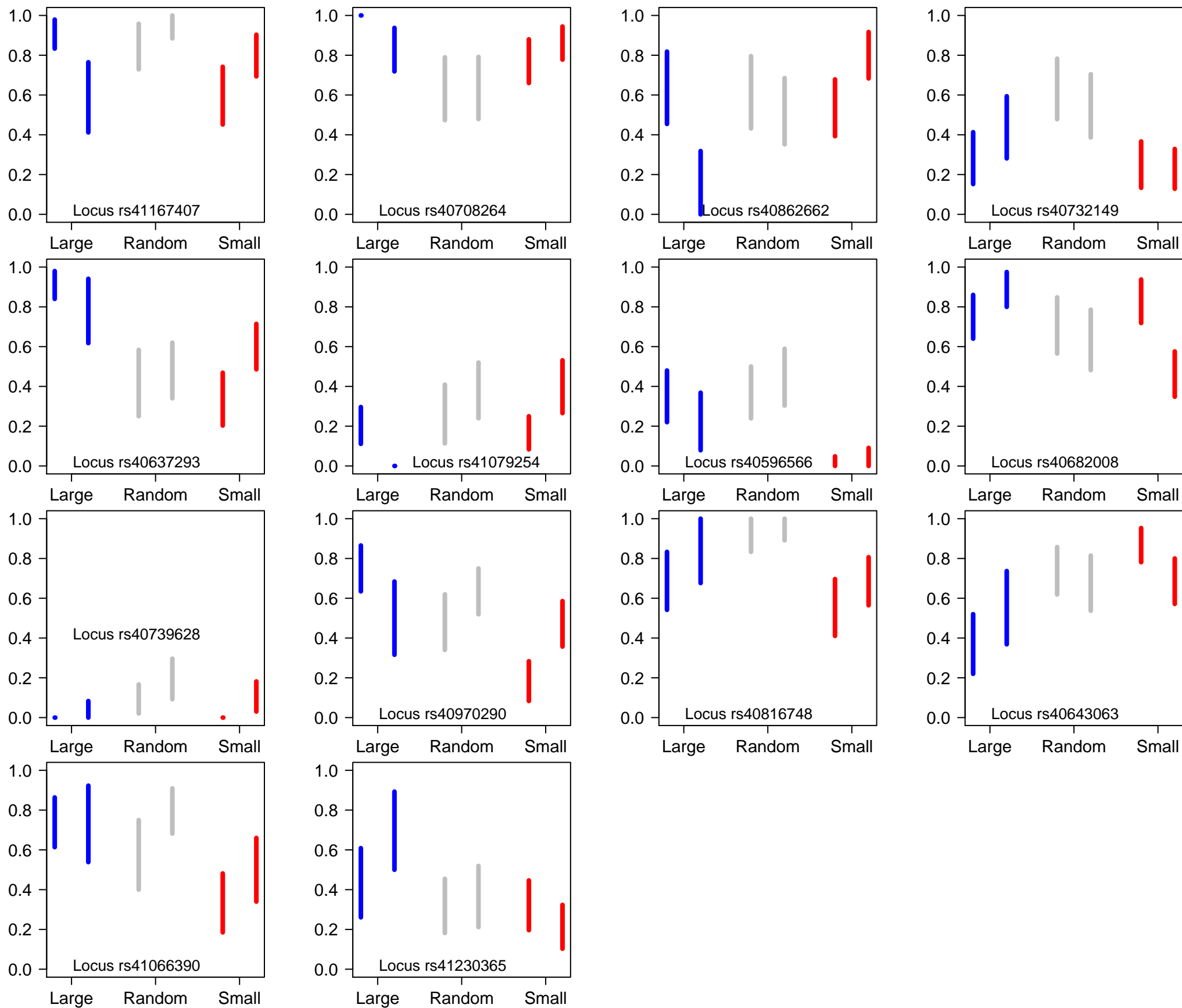

Supplement: Supplementary file 1 [file eva0008-0597-sd1.pdf]

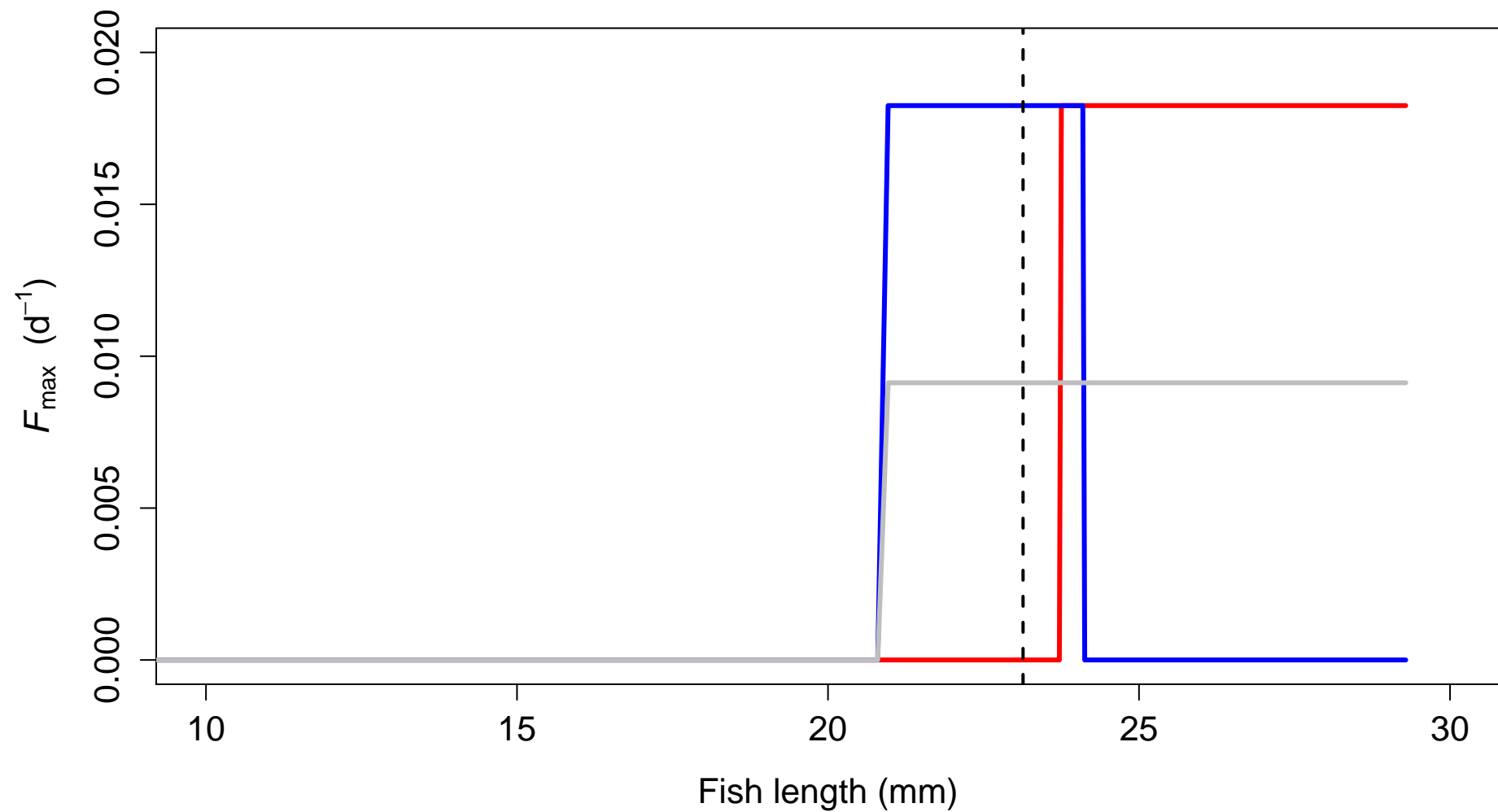

Supplement: Supplementary file 2 [file eva0008-0597-sd2.pdf]

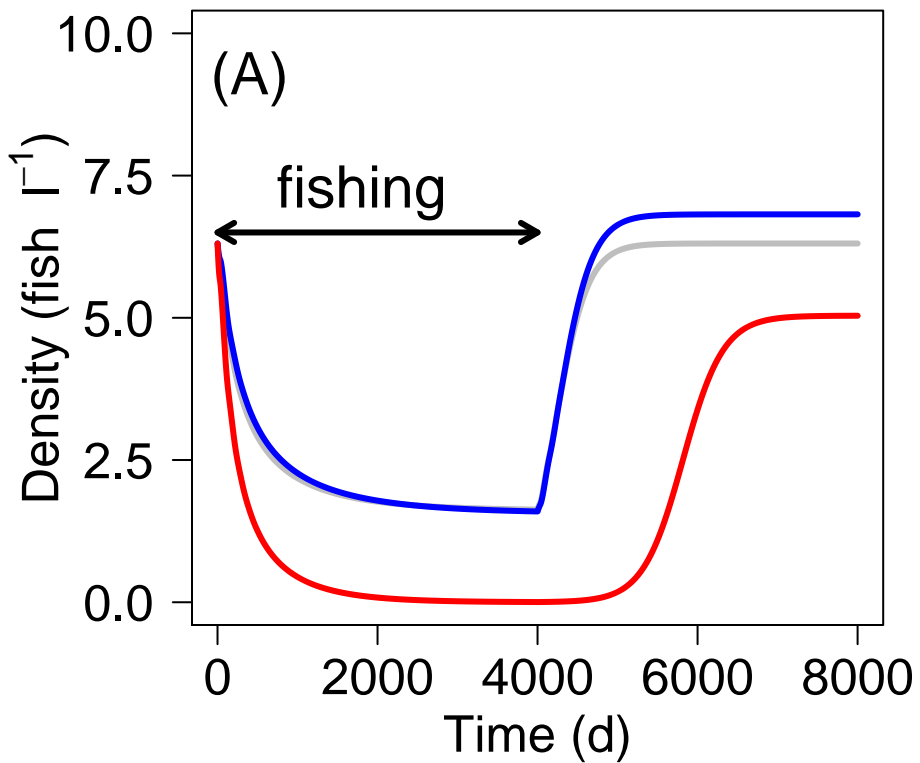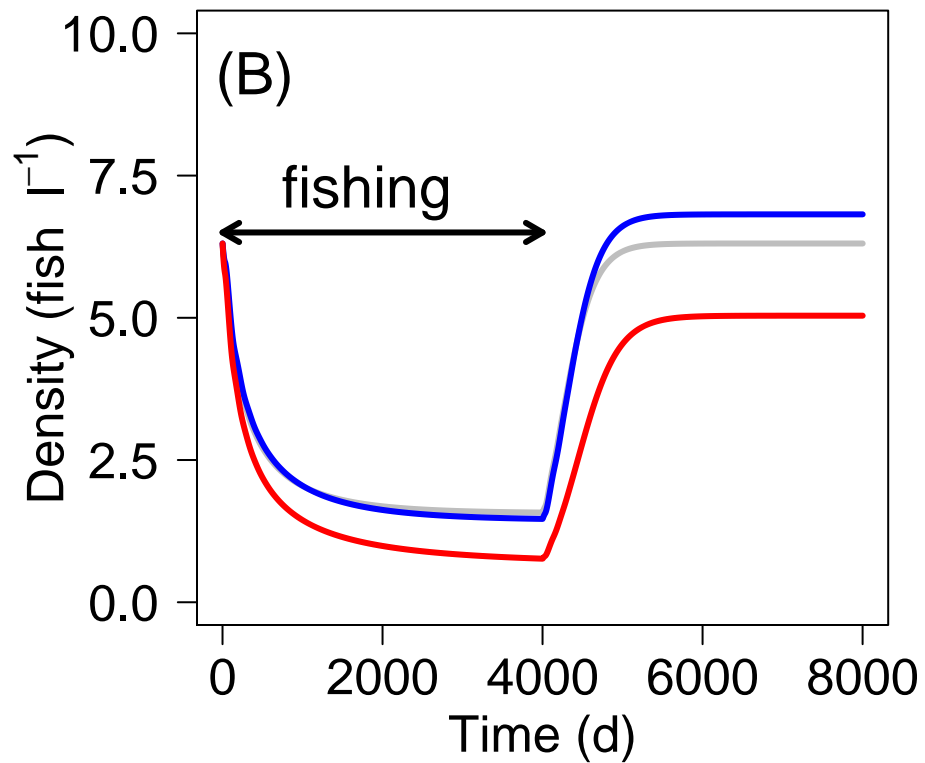

Supplement: Supplementary file 3 [file eva0008-0597-sd3.pdf]

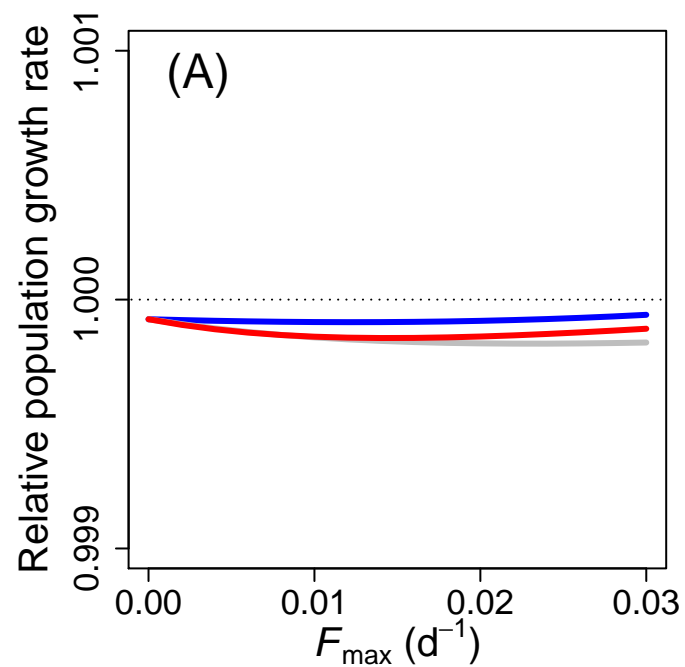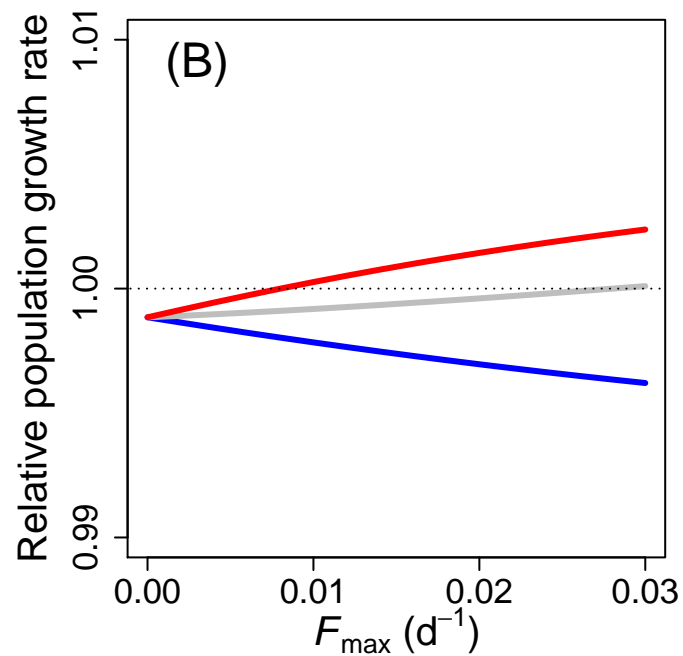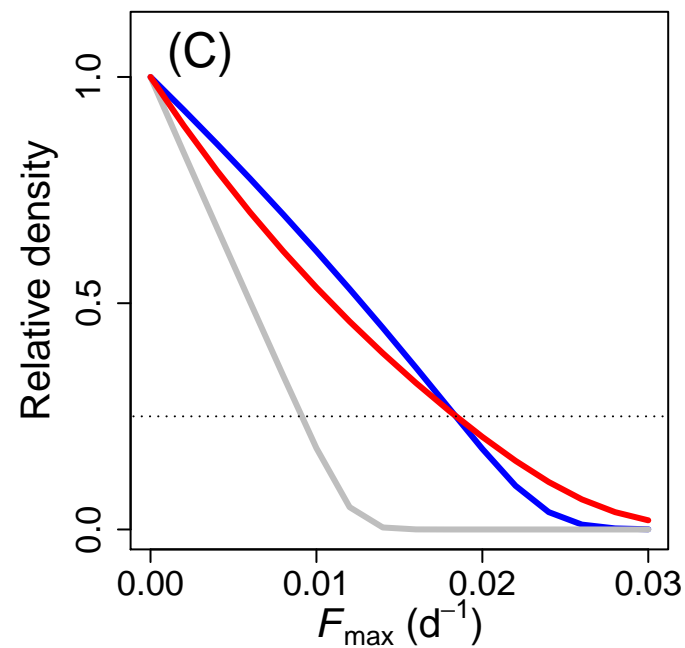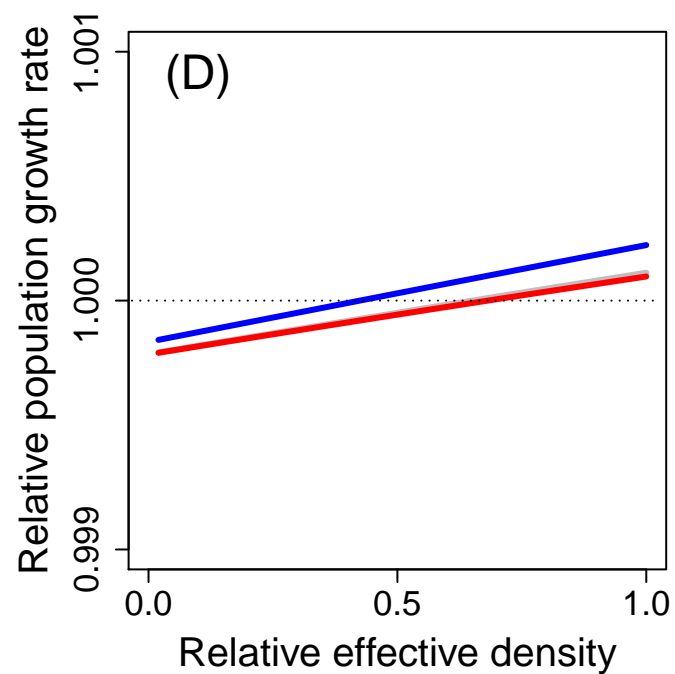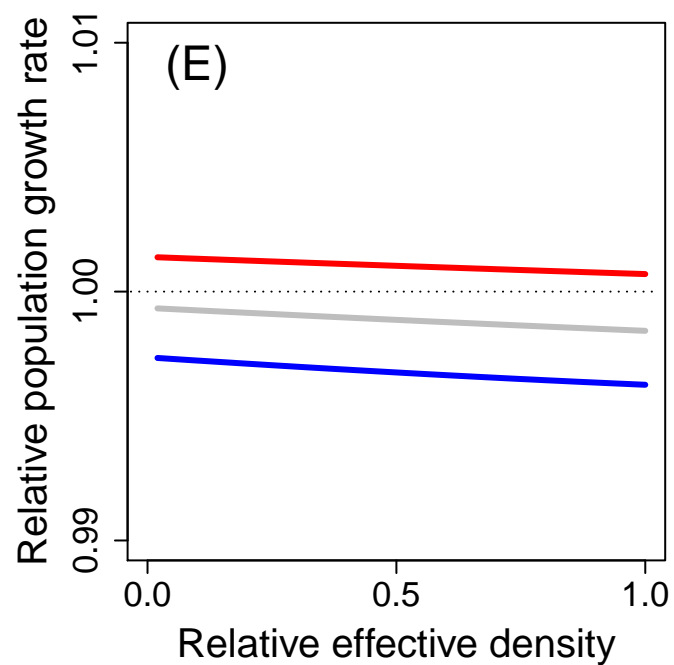

Supplement: Supplementary file 4 [file eva0008-0597-sd4.pdf]
