# Supplementary material for: The evolutionary legacy of size-selective harvesting extends from genes to populations
Source: Evol Appl. 2015 May 27;8(6):597–620. doi: 10.1111/eva.12268 (PMC4479515; doi:10.1111/eva.12268)
Supplement: Supplementary file 5 [file eva0008-0597-sd5.docx]

**SUPPORTING INFORMATION**

**METHODS AND RESULTS**

**Zebrafish rearing conditions – S1**

We used wild zebrafish in our experiment to ensure maximum genetic variation (heterozygosity across 384 single nucleotide polymorphisms was 0.42, n = 60) and to minimize the possibility that the fish had previously experienced domestication selection or inbreeding. Throughout the rearing phase for each generation, all six populations (three selection lines with two replicates each, N = 450 per replicate) were maintained in separate tanks (volume 320 l) in a light-dark (14L:10D) and temperature-controlled (mean ± sd, 27.01 °C ± 1.23 °C) recirculation facility with an inflow rate of 0.25 l s^-1^. The recirculation system was run with tap water and the water quality was controlled weekly for pH (8.41 ± 0.11), nitrite (< 0.3 mg l^-1^) and ammonium (< 0.05 mg l^-1^), and daily for oxygen levels (8.00 ± 0.21 mg l^-1^). The stocking density per holding tank was 0.9 ± 0.2 individuals l^-1^. All individuals were reared in identical densities to control for density effects and to minimize the potential bias of dominance hierarchies on feeding success. Fish were fed *ad libitum* five times a day with *Artemia* nauplii and commercial flake food (TetraMin; 47 % protein, 10 % fat). Hatchlings were fed *ad libitum* seven times per day with commercial food for larval fish (Sera Micron, 52 % protein, 7 % fat) until they were large enough to feed *Artemia* nauplii (at age 15 d).

**Life-time growth and analysis of life-history traits – S2**

*Methods*

To estimate the evolutionary response of life-time growth under *ad libitum* feeding, we stocked F_9_-generation offspring (selection halted for three generations) from each selection line into three liter rearing boxes at age 20 days (d). The density was standardized to 10 individuals per box (0.3 individuals l^-1^). We established 20 replicate boxes for the large fish, 20 boxes for the small fish and 20 boxes for the random fish. However, only 16 replicate boxes for the large, 14 for the small, and 18 for the random fish were used when modeling growth because the remaining replicate boxes experienced high rates of handling mortality and / or fish escapees. We measured the fish every ~15 d between 30 and 210 d of age. We could not accurately measure fish larvae younger than 30 d without causing injury or death. Growth data were combined for sexes because we could not determine sex noninvasively in juveniles. Combined sexes should not bias our growth estimates because of low sexual dimorphism in zebrafish body size (Spence and Smith 2006).

To estimate key life-history traits, we used a function-valued trait approach to infer juvenile growth rate (*h*), reproductive investment (*g*) and maximum asymptotic length (*L_∞_*) from treatment- and replicate-specific growth curves. To this end, we applied the biphasic growth model (Lester et al. 2004) to length-at-age data (females and males combined) in each experimental population. The biphasic growth model comprises two functions that together account for the change in growth that occurs with maturation (i.e., investment in reproduction, *g*): a linear function that describes immature growth, and a widely-used von Bertalanffy (asymptotic) function that describes adult growth. Because we did not know the age (*T*) at which fish in each selection line began to invest in reproduction, we fitted the biphasic model to all integer values of *T* over a plausible range and then selected the fit that minimized the residual sum of squares. For each integer value of *T*, we estimated all other life-history parameters as follows. First, we fitted a linear model to the supposed immature data points to estimate immature growth rate (*h*; mm d^-1^) and the x-intercept (*t_1_*; d). We then used published equations (Lester et al. 2004) to predict i) daily instantaneous mortality rate (*M*) from *T* and *t_1_*, ii) daily investment in reproduction (*g*) from *M*; iii) asymptotic length (*L_∞_*; mm) from *h* and *g*; iv) the exponential rate at which the average individual approaches *L_∞_* (*k*) from *g*; and v) the x-intercept of adult growth (*t_0_*) from *g*, *T*, and *t_1_*. This approach to predicting life-history traits from model parameters assumed that life-history parameters in each selection line were optimally adapted to treatment conditions.

We used the output from the biphasic growth model to test for evolved differences in life-history traits (*h*, *g*, *T*, *L_∞_* and *M*) among the selection lines. The response variable in each analysis was the average, replicate box-specific value predicted by the biphasic growth model. For all statistical analyses, we used linear mixed-effects model and set selection line as a predictive variable and selection line replicate as a random variable.

*Literature*

Lester, N. P., B. J. Shuter, and P. A. Abrams 2004. Interpreting the von Bertalanffy model of somatic growth in fishes: the cost of reproduction. Proceedings of the Royal Society B: Biological Sciences **271**:1625–31. doi:10.1098/rspb.2004.2778.

Spence, R., and C. Smith. 2006. Mating preference of female zebrafish, *Danio rerio*, in relation to male dominance. Behavioral Ecology **17**: 779–83. doi:10.1093/beheco/arl016.

**Probabilistic maturation reaction norm (PMRN) – S3**

*Methods*

To estimate PMRNs for each selection line, we conducted a maturation experiment, similar to our previous work (Uusi-Heikkilä et al. 2011), using offspring from the F_8_-generation (selection halted for two generations). The larvae were reared in the five liter rearing boxes for the first 20 d after which they were transferred into 45 glass aquaria (volume 45 l) in a recirculation holding facility. We established five replicate aquaria per selection line per diet. Fish were reared in equal densities (1.3 individuals per liter) and environmental conditions until age 85 d, after which we induced variation in growth and maturation by applying one of three feeding regimes: 1 %, 2 % and 4 % dry food of the body weight daily. Thereby, we “sampled” fish from different portions of the probabilistic maturation reaction norm (PMRN) to derive the full plasticity in maturation timing as a function of growth variation. Fish were exposed to these diets until age 190 d. Every 10 d we lethally assessed the SL and maturation status of approximately 15 randomly sampled females from each aquarium (Uusi-Heikkilä et al. 2011).

To estimate the PMRNs, we used the demographic estimation method (Barot et al. 2004), which involves estimation of maturity ogives, modeling growth rates and estimation of the PMRNs based on the maturity ogives and growth model. We estimated the maturity ogives for each selection line (treatment replicates were pooled because they did not differ significantly from one another) across all diets by including age, length, and relative condition factor (Uusi-Heikkilä et al. 2011) as predictor variables and diet as a random variable:

*o(a, l, c, d) ~ a + l + c + d*

In the ogive model (*o*), age (*a*) in days, standard length (*l*) in mm and relative condition factor (*c*) were continuous variables and the random effect, diet (*d*), was a categorical variable. The model was additionally tested with the quadratic terms of age (*a^2^*), length (*l^2^*) and relative condition factor (*c^2^*) and with the interactions of age, length and relative condition.

Growth in length was modeled with linear multiple regressions with age as a predictor variable and diet as a random variable. Relative condition was modeled similarly by using age, length and their interactions as predictor variables, and diet as a random variable. In growth and condition models, the quadratic and cubic terms of age (*a^2^*, *a^3^*) and length (*l^2^*, *l^3^*) were added to the model to test the non-linearity of the predicted relationships.

The PMRNs were constructed for each selection line separately using the following estimation model:

*m(a, l, c)* = *o(a, l, c, d) – o(a[i] – a[i – 1], l – Δl, c - Δc) / 1 – o(a[i] – a[i – 1], l – Δl, c – Δc)*,

where *m* refers to the probability of maturing. It was calculated from the maturity ogive, *o(a, l, c, d)* and the mean age-specific growth increments in length (*Δl*) and the mean age-specific changes in relative condition (*Δc*), which were included from the growth and condition models (Uusi-Heikkilä et al. 2011).

*Results*

During the maturation experiment, independent of the diet to which the fish were exposed in order to induce environmental differences in growth, 19.7 % of the small-selected fish matured (58 females), whereas 44.9 % (106 females) of the large-selected and 28.4 % of the random fish (74 females) matured. When pooled across the diets and the experimental period of the maturation experiment (104–186 d), the small-selected fish were on average smaller (17.1 ± 0.17 mm; mean ± SE) than the large-selected (19.3 ± 0.21 mm) and random fish (18.6 ± 0.20 mm), and the small-selected fish also had lower relative condition factor (1.042 ± 0.026) compared to the random (1.097 ± 0.021) and large-selected fish (1.092 ± 0.037).

Unlike among the small-selected and random fish, the growth of the large-selected fish was not linear. This was indicated by the significant effect of quadratic and cubic terms of age (*a^2^*, *a^3^*) in the growth model (Table S1). The relative condition factor of the large-selected fish decreased with length and was not affected by age (Table S1), whereas among the small-selected and random fish the relative condition seemed to be affected by both age and length (indicated by the significant age × length interaction; Table S1).

Table S1. Final growth and condition models with their covariates and *t*-statistics for each selection line.

| **GROWTH** | | | |
| --- | --- | --- | --- |
| **Selection line**  Large  Random  Small | **Variable**  Age  Age^2^  Age^3^  Age  Age | ***t*-value (df)**  -1.644 (230)  1.844 (230)  -1.980 (230)  6.670 (257)  7.227 (290) | ***P*-value*^a^***  0.1016  0.0665  0.0489  <0.0001  <0.0001 |
| **RELATIVE CONDITION** | |  |  |
| Large  Random  Small | Length  Age  Length  Age × Length  Age  Length  Age × Length | -2.014 (232)  -3.643 (255)  -5.637 (255)  3.752 (255)  -3.167 (288)  -4.125 (288)  3.207 (288) | 0.0452  <0.0001  <0.0001  <0.0001  0.0017  <0.0001  0.0015 |

**^a^** Derived from the linear mixed-effects model fit by maximum likelihood.

Table S2. Maturity ogive models for each selection line.

| **Selection line** | **Variable** | **χ^2^-value^a^ (df)** | ***P*-value*^b^*** |
| --- | --- | --- | --- |
| Large  Random  Small | Age  Length  Condition  Age  Length  Condition  Age  Length  Condition | 11.768 (4,5)  38.420 (4,5)  0.7700 (4,5)  33.864 (4,5)  24.316 (4,5)  7.1502 (4,5)  7.1676 (4,5)  41.381 (4,5)  15.505 (4,5) | 0.0006  <0.0001  0.3802  <0.0001  <0.0001  0.0075  0.0074  <0.0001  <0.0001 |

^a^ χ^2^-value from the deletion of the variable from the full model.

^b^ *P*-values derived from the χ^2^ –statistics.

*Literature*

Barot, S., M. Heino, L. O’Brien, and U. Dieckmann 2004. Estimating reaction norms for age and size at maturation when age at first reproduction is unknown. Evolutionary Ecology Research **6**:659–78.

Uusi-Heikkilä, S., A. Kuparinen, C. Wolter, T. Meinelt, A. C. O'Toole, and R. Arlinghaus 2011. Experimental assessment of the probabilistic maturation reaction norm: condition matters. Proceedings of the Royal Society B - Biological Sciences **278**:709–17. doi:10.1098/rspb.2010.1507.

**Reproductive performance and early life-history traits – S4**

*Methods*

We estimated the reproductive performance of zebrafish in each selection line for the F_9_-generation fish (selection halted for three generations). At age 230 d, one female and one male from each of the selection line replicate, whose SL and WM were first assessed, were stocked into one three-liter spawning box for five days (altogether 20 spawning boxes per treatment). To estimate reproductive output, we assessed daily spawning frequency and clutch size (i.e., the number of eggs spawned per female per spawning event). To estimate the egg fertilization rate, fertilized eggs were separated from the unfertilized eggs. We used a previously published protocol (Uusi-Heikkilä et al. 2012) to estimate egg size (measured as yolk diameter), egg survival, hatching probability, larval age-at-hatch, larval length-at–hatch, and larval yolk-sac volume. The probability of successful swim bladder inflation was estimated from seven days old larvae, which were successfully hatched from 25 fertilized eggs placed in a Petri dish and kept in the incubator. To estimate larval survival, 25 fertilized, healthy eggs (i.e., not suffered from egg mortality at age 4 d) were placed in separate Petri dishes, and after hatching the larvae were stocked into three-liter rearing boxes and fed *ad libitum*. At age 10 d the number of survived larvae in each rearing box was calculated.

All statistical analyses on the early life-history traits were performed using a generalized linear model to account for the non-normally distributed and heteroscedastic data (Crawley 2007). If data were over- or under-dispersed, the dispersion parameter was defined in the model. To estimate differences in reproductive performance and in early life-history traits among selection lines, we first fitted the full model and then used the stepwise model reduction procedure (Crawley 2007). In all of the statistical analyses, selection line was treated as a predictive variable and both selection line replicate, spawning day (when estimating the effects of selection on variables measured over a five day spawning trial, i.e., spawning probability, clutch size and egg fertilization probability) and spawning couple were treated as random variables. To study whether other factors related to selection, except female and male body size, could have affected the variation in reproductive traits among the selection lines, we analyzed the residual variation not accounted by the female and male body size as a function of the selection treatment. In the final analyses, however, the residual variation in the reproductive and early life-history traits (i.e., variation not explained by the female and male body size) was unrelated to the selection treatments.

*Literature*

Crawley, M.J. 2007. The R Book. West-Sussex. Wiley.

Uusi-Heikkilä, S., A. Kuparinen, C. Wolter, T. Meinelt, and R. Arlinghaus 2012. Paternal body size affects reproductive success in laboratory-held zebrafish (*Danio rerio*). Environmental Biology of Fishes **93**:461–74. doi:10.1007/s10641-011-9937-5.

**Metabolic rate and exploration – S5**

*Methods*

Juvenile zebrafish of the F_10_-generation, i.e., when selection had been halted for four generations, (mean ± s.d. length and weight of small-selected fish: 21.9 ± 2.85 mm and 0.108 ± 0.039 g [N = 42], random fish: 22.0 ± 3.48 mm and 0.104 ± 0.046 g [N = 39] and large-selected fish: 21.8 ± 3.42 mm and 0.096 ± 0.044 [N = 41]) were fasted for 24 h and were then transferred into one of seven individual 4 ml glass respirometers (Loligo Systems, Tjele, Denmark). Respirometers were immersed in a water bath that was maintained at a constant temperature or 25 ± 0.1 ^o^C. The water surface was shielded with opaque polystyrene to prevent visually disturbing the fish, and all disturbances were kept to a minimum during measurements. After a two hour acclimation period, standard metabolic rate (SMR) was measured as rates of oxygen uptake using intermittent stopped-flow respirometry (Steffensen 1989; Killen et al. 2011). Pilot trials determined that two hours was sufficient time for the fish to settle, as rates of oxygen uptake did not decline further with time beyond this point. Water flow from the external bath through the respirometers was driven by an external pump that was set to turn on and off for alternating 15 min periods. This allowed decreases in water oxygen content to be measured every two seconds for 15 min while the respirometer was in the closed state, and then the respirometer was flushed with aerated water for 15 min. The oxygen consumption during each closed phase was calculated using linear least squares regression (excluding the first and last two minutes of each closed phase). An eighth respirometer was left empty, to provide parallel measurements of background microbial respiration in the system. Water oxygen levels were measured with optodes (Firesting 4-Channel oxygen meters; Pyroscience, Germany), and oxygen uptake for each measurement period was then calculated according to previously published protocol (Dupont-Prinet et al. 2010). For each individual, six slopes of oxygen decrease were recorded, and SMR was taken as the mean of these measures. Rates of oxygen consumption were corrected for differences in body size by taking the residual of oxygen uptake (mg h^-1^) versus body mass (g) for each individual, then adding this fitted value of this relationship at 0.1 g (the approximate mean mass of all fish used in the study) (Killen et al. 2010). Differences in SMR among treatments were examined using a linear mixed model, with SMR at 0.1 g as the dependent variable, selection line as a categorical fixed effect, and selection line replicate as a random effect.

After testing for metabolic rate, we transferred test fish (19 small fish, 22 random fish and 27 large fish) to the five liter rearing boxes and let them acclimatize overnight. The following day, each individual was tested twice (with a break of 30 min between measurements) for its exploration behavior in an open field test in a novel environment (Ariyomo and Watt 2012). Exploration of a novel, open field environment has been a useful measure of an individual’s position in the shy-boldness continuum in teleost fishes (Burns 2008; Ariyomo and Watt 2012, 2013; Carter et al. 2013). The open field arena consisted of a circular tank (48.5 cm in diameter, made of white plastic). We used system water to fill the arena to a depth of 7 cm. Lighting was provided from two neon tubes positioned at the room ceiling, which helped to avoid shadows or reflections within the tank. In each trial, a single fish was introduced into a transparent plastic cylinder in the center of the arena and it was let to acclimate for five minutes. Afterwards, the cylinder was carefully removed and the fish movement was videotaped with a webcam for the next five minutes (1^st^ trial). After that, we transferred the test fish back into the rearing box. Measurements were repeated for each individual after a break of 30 minutes (2^nd^ trial). Videos were analyzed using the video tracking software EthoVision XT Version 9.0 (Noldus Information Technologies, Inc.). Position scoring started 10 s after the fish was released from the cylinder. Altogether we analyzed 250 s per individual and per trial.

When analyzing the data, we first searched for correlation between both response variables (i.e., velocity and time spent freezing) using a principle component analysis (PCA) for both test trials. The first principal component (PC1) accounted for 93.8% of the total variance and both variables loaded with 0.97 on this component (velocity in the negative direction and time spent freezing in the positive direction). Due to this high, negative correlation, both variables were likely to represent the same personality trait (i.e., boldness; Dahlbom et al. 2014). Both variables were square-root transformed prior to all analyses to approach Gaussian error distribution. We used the first principal component (PC1) as a response variable in a linear mixed model in which selection treatment, experimental trial, TL, and WM were predictive variables and individual and selection line replicate were random variables. Model fitting was performed by first evaluating the random effects through likelihood ratio tests. We then excluded all covariates with *P* > 0.1 and refitted the model. In the final model, all other explanatory variables could be excluded, except the selection treatment (predictive variable) and the individual (random variable). We omitted selection line replicate from the final model as this random effect was not significant (*χ*²=1.96, *df*= 1, *P*=0.27) and only explained 8.7 % of the total phenotypic variance. Based on the obtained variance of the residual error (“within individual variance”) and the random intercept variance (“between individual variance”), we calculated the repeatability for PC1 (Dingemanse and Dochtermann 2013).

*Results*

Table S3. Differences in PC1 score (estimated marginal means) derived from the final model among the selection lines. Note that increasing PC1 score implies less explorative individuals and individuals that freeze for a longer duration.

| **Selection line** | **Estimated value** | **SE** | **Confidence intervals** |
| --- | --- | --- | --- |
| Large  Random  Small | -0.350  0.293  0.163 | 0.163  0.179  0.193 | -0.676 – -0.023  -0.064 – 0.650  -0.222 – 0.547 |

*Literature*

Ariyomo, T. O., and P. J. Watt 2013. Disassortative mating for boldness decreases reproductive success in the guppy. Behavioral Ecology **24**:1320–26. doi:10.1093/beheco/art070.

Ariyomo, T. O., and P. J. Watt 2012. The effect of variation in boldness and aggressiveness on the reproductive success of zebrafish. Animal Behaviour **83**:41–46. doi:10.1016/j.anbehav.2011.10.004.

Burns, J. G. 2008. The validity of three tests of temperament in guppies (*Poecilia reticulata*). Journal of Comparative Psychology **122**:344–56. doi:10.1037/0735-7036.122.4.344.

Carter, A. J., W. E. Feeney, H. H. Marshall, G. Cowlishaw, and R. Heinsohn 2013. Animal personality: what are behavioural ecologists measuring? Biological Reviews **88**:465–75. doi:10.1111/brv.12007.

Dahlbom, S. J., D. Lagman, K. Lundstedt-Enkel, L. Fredrik Sundström, and S. Winberg 2014. Boldness predicts social status in zebrafish (*Danio rerio*). PloS One **9**:e23565. doi:10.1371/journal.pone/0023565.

Dingemanse, N. J., and N. A. Dochtermann 2013. Quantifying individual variation in behaviour: mixed-effect modelling approaches. The Journal of Animal Ecology **82**:39–54. doi:10.1111/1365-2656.12013.

Dupont-Prinet, A., B. Chatain, L. Grima, M. Vandeputte, G. Claireaux, and D. J. McKenzie 2010. Physiological mechanisms underlying a trade-off between growth rate and tolerance of feed deprivation in the European sea bass (*Dicentrarchus labrax*). The Journal of Experimental Biology **213**:1143–52. doi:10.1242/jeb.037812.

Killen, S. S., D. Atkinson, and D. S. Glazier 2010. The intraspecific scaling of metabolic rate with body mass in fishes depends on lifestyle and temperature. Ecology Letters **13**:184–93. doi:10.1111/j.1461-0248.2009.01415.x.

Killen, S .S., S. Marras, and D. J. McKenzie 2011. Fuel, fasting, fear: routine metabolic rate and food deprivation exert synergistic effects on risk-taking in individual juvenile European sea bass. The Journal of Animal Ecology **80**:1024–33. doi:10.1111/j.1365-2656.2011.01844.x.

Steffensen, J. F. 1989. Some errors in respirometry of aquatic breathers: how to avoid and correct for them. Fish Physiology and Biochemistry **6**:49–59. doi:10.1007/BF02995809.

**Genetic analyses – S6**

*Methods*

We extracted DNA from the muscle tissue of fish from the founder population, F_2_ –generation, F_4_ –generation and F_7_ –generation, one generation after selection was halted. We sampled 60 fish (females and males) from the founder population and 30 fish from each selection-line replicate per generation. From the control treatment, only fish from the F_2_-and F_7_ -generations were used for the genetic analyses. The DNA was extracted with the QIAGEN DNeasy Blood & Tissue Kit ([www.qiagen.com](http://www.qiagen.com)) following the manufacturer’s instructions. Samples were genotyped according to the manufacturer’s protocol on an Illumina BeadXpress VeraCode genotyping platform ([www.illumina.com](http://www.illumina.com)) using a custom panel. We examined variation at 371 single nucleotide polymorphisms (SNPs), which resulted in an average of 14.84 markers per chromosome with an average distance of 3.91 Mb. Map positions of the markers were based on map locations from the Zv9 zebrafish genome assembly.

To determine if size-selective harvesting had induced genetic changes in the experimentally exploited zebrafish populations, we used 384 genome-wide, evenly distributed single nucleotide polymorphisms (SNPs), that were chosen from a previously analyzed wild zebrafish dataset (Whiteley et al. 2011). We had to exclude 38 individuals due to the low genotyping success. We excluded eight markers that were not successfully genotyped for ≥ 50 % of the individuals. Five additional markers deviated from Hardy-Weinberg proportions and were excluded. Thus, we examined variation at 371 markers in 502 individuals. The outlier analysis was conducted for F_7_ –generation individuals using the FDist method (Beaumont and Nichols 1996) implemented in software LOSITAN – Selection Workbench (Antao et al. 2008). The outlier analysis identifies outlier loci as those that show significantly higher F_ST_ than expected by processes other than selection. LOSITAN estimated outlier loci responding significantly to divergent and balancing selection from the entire dataset of 371 SNPs. We used an infinite alleles model and 50 000 simulations.

We also tested for linkage disequilibrium (LD), or non-random association among loci. LD is affected by several factors including genetic drift and selection, which can, for example create LD between genes by favoring specific gene combinations. In our study, any outlier SNPs that were located on the same chromosome were tested for LD to establish whether these outliers were likely to be associated with multiple sites under selection. Low LD indicates that same SNPs are associated with different sites of selection whereas high LD could suggest that the same SNPs are associated with the same site. We tested each pair of loci in each population for LD using the log likelihood ratio statistics in Genepop version 4.2. The *P*-values were adjusted to multiple testing with a Bonferroni correction.

*Results*

Two pairs of the outlier loci on chromosomes 9 (physically separated by 11,049 kb) and 10 (separated by 15,804 kb) were in significant linkage disequilibrium.

Table S4. The SNP name, chromosome location, map position (Mb), F_ST_-value, heterozygosity and the adjusted *P*-value (for empirical F_ST_ higher than simulated F_ST_, false discovery rate set at 0.1, at 95 % confidence interval level) of the outlier loci detected using the FDist method.

| **SNP name^a^** | **Chromosome** | **Map position** | **F_ST_** | **Heterozygosity** | ***P*-value** |
| --- | --- | --- | --- | --- | --- |
| rs41167407  rs40643063  rs41161981  rs40907508  rs40708264  rs40862662 | NA  NA  5  5  6  8  9  9  10  10  10  11  12  12  15  18  18  18  20  21  23  25 | NA  NA  23.897889  50.455574  37.424300  27.737846  43.806552  54.855562  20.181385  35.985107  52.684650  26.546278  31.415453  38.182275  42.367350  16.649014  21.512397  44.400741  33.520900  65.792310  32.275844  37.574871 | 0.130  0.115  0.150  0.114  0.122  0.144  0.125  0.129  0.104  0.115  0.193  0.125  0.123  0.125  0.142  0.152  0.117  0.109  0.173  0.104  0.166  0.133 | 0.440  0.466  0.510  0.481  0.347  0.504  0.487  0.351  0.487  0.479  0.501  0.431  0.511  0.512  0.485  0.373  0.406  0.485  0.513  0.486  0.322  0.512 | 0.005  0.018  0.005  0.019  0.014  0.004  0.013  0.010  0.024  0.018  0.0002  0.008  0.017  0.016  0.006  0.003  0.019  0.024  0.002  0.024  0.002  0.011 |
| rs40732149**^*^**  rs40816748**^*^**  rs41230365**^*^**  rs41079254**^*^**  rs40637293  rs40865556  rs40596566  rs41115354  rs40856687  rs40688310  rs40682008  rs41066390  rs40739628  rs41238352  rs40655095  rs40970290 |

**^a^** An asterisk indicates the two outlier SNPs in chromosomes nine and ten that were in significant LD.

Table S5. Outlier loci responding to balancing selection: type of the variant (UTR = untranslated region, D = downstream gene variant, I = inter-genic variant, In = intron variant), gene name, and gene function. Loci with parallel and consistent allele frequency changes (i.e., decrease or increase in all lines between the founder population and F_7_-generation) possibly supporting the fecundity selection hypothesis (see Discussion in the main text) are indicated in bold.

| **Outlier SNP name** | **Chromosome** | **Map position** | **Type** | **Gene name** | **Function** |
| --- | --- | --- | --- | --- | --- |
| rs40792992  **rs40624233**  rs40656596  rs41214240  rs41140266  rs40627378  **rs40620845**  rs41189461  rs40786572  **rs40870101**  rs40967394  rs40918563 | 1  **1**  2  3  3  6  **13**  14  16  **18**  18  20 | 14347199  **17337934**  21266167  41700947  46836905  15427030  **11159374**  48572107  27836773  **6817729**  12771702  38751644 | In  **UTR**  UTR  UTR  D  UTR  **D**  I  NS  **NS**  UTR  UTR | SGCZ  **klf3**  afg3l2  gna12a  elavl3  irrfip1b  **zbtb18**  No description available.  No description available.  **glipr1b**  plcg2  uts1 | Part of the sacroglycan complex.  Interacting selectively and non-covalently with any metal ion.  **Metal ion binding** (Varshney et al. 2013).  Proteolysis.  Cell migration involved in gastrulation (Lin et al. 2005).  RNA binding (Ahrens et al. 2012).  No description available.  **Encodes a zinc finger protein involved in neuronal development.**  No description available.  No description available.  **Function not characterized in zebrafish.**  Intracellular signal transduction.  Hormone activity (glycopeptide and lipopeptide) and metal ion binding (Alderman and Bernier 2007). |

Table S6. Outlier loci with the most pronounced, replicable allele frequency divergence among selection treatments. Also shown are the type of the variant (S = synonymous, UTR = untranslated region, D = downstream gene variant, I = inter-genic variant, In = intron variant), gene name, and gene function.

| **SNP name** | **Treatment** | **Type** | **Gene name** | | **Gene function** |
| --- | --- | --- | --- | --- | --- |
| rs41161981  rs40907508  rs40865556 | Large ≠ small  Large ≠ small  Large ≠ small and random | D  UTR  S | las1l  LysM  ccdc120 | | Function not identified in zebrafish.  The chemical reactions and pathways resulting in the breakdown of macromolecules that form part of a cell wall.  Function not formally identified. |
| rs41115354  rs40856687  rs40688310 | Large ≠ small  Large ≠ small  Large and random ≠ small | I  In  UTR | No description available.  No description available. | | |
|  |  |  | Tryptophan hydroxylase 2 | | The chemical reactions and pathways involving aromatic amino acid family. Controls brain serotonin synthesis in human and mice. |
| rs41238352  rs40655095 | Large ≠ small  Large ≠ small | In  S | col5a1 | Function not identified in zebrafish. | |
|  |  |  | eph receptor B2b | | The process of introducing a phosphate group on to a protein. Regulates transcription. |

Table S7. Outlier SNPs that were in linkage disequilibrium (LD) with a nearby SNP (LD SNP) and the physical distance in kilobase pairs between them. The gene information, i.e., the type of the variant (UTR = untranslated region, S = synonymous, D = downstream gene variant, U = upstream gene variant, In = intron variant, I = inter-genic variant, NS = non synonymous variant), the name and the function of the in which the LD SNP occurs.

| **Outlier SNP name** | **LD SNP name** | **Genetic distance (kbp)** | **Type** | **Gene name** | **Function** |
| --- | --- | --- | --- | --- | --- |
| **rs41161981** | rs41021709  rs41092660  rs41141381 | 180  19 210  22 531 | UTR  S  UTR | GAB3  eef2l2  Cathepsin L 1 a | Function not identified in zebrafish.  Interacting selectively and non-covalently with guanosine triphosphate.  Involved in embryonic yolk processing (Tingaud-Sequeira and Cerdà 2007). |
| **rs40907508** | rs41141381  rs40964259  rs40627745  rs40622039  rs40933327 | 4 026  5 331  17 226  18 139  30 864 | UTR  D  D  U  UTR | Cathepsin L 1 a  rhobtb2a  MEGF9  pdzd3b  Checkpoint with forkhead and ring finger domains, E3 ubiquitin protein ligase | Involved in embryonic yolk processing (Tingaud-Sequeira and Cerdà 2007).  Involved in small GTPase-mediated signal transduction.  Function not identified in zebrafish.  Interacting selectively and non-covalently with ATP.  Cell division, mitosis, protein binding. |
| **rs40865556** | rs40895761  rs40784742  rs40910234 | 6 843  7 889  8 861 | D  UTR  In | dnmbp  lims1  tmem180 | Function not identified in zebrafish.  Involved in cardiac contraction and heart beating.  Function not identified in zebrafish. |
|  | rs41057458  rs40687123 | 11 633  22 624 | I  S | No description available. | |
|  |  |  |  | TANK2 | Function not identified in zebrafish. |
| **rs40856687** | rs40769175  rs41052587 | 1 356  9 328 | UTR | Guanylate cyclase activator 1C | Involved in calcium signaling in the eye and in light regulation, circadian rhythms and stress response (Scholten and Koch 2011; Weger et al. 2011). |
|  |  |  |  | No description available. | |
| **rs41238352** | rs40739415 | 7 335 | D | ddt | Involved in inner ear development. |
| **rs40655095** | rs40668802  rs40746508  rs40878095 | 5 258  7 319  31 390 | S  UTR  NS | galnt6  CTD small phosphatase 2  Interleukin-1 receptor-associated kinase 4 | Involved in protein glycosylation.  Involved in neuron differentiation.  Involved in innate immune response system in zebrafish (Stein et al. 2007). |

*Supporting figure legends*

Figure S1. Outlier loci, which showed no replicable (consistent) response to selection but were detected by the outlier test (FDist method). Large fish indicated with blue lines, random fish with gray lines, and small fish with red lines.

*Literature*

Ahrens, M. B., J. M. Li, M. B. Orger, D. N. Robson, A. F. Schier, F. Engert, and R. Portugues 2012. Brain-wide neuronal dynamics during motor adaptation in zebrafish. Nature **485**:471–77. doi:10.1038/nature11057.

Alderman, S. L., and N. J. Bernier 2007. Localization of corticotropin-releasing factor, urotensin I, and CRF-binding protein gene expression in the brain of the zebrafish, Danio rerio. The Journal of Comparative Neurology **502**:783–93. doi:10.1002/cne.21332.

Antao, T., A. Lopes, R. J. Lopes, A. Beja-Pereira, and G. Luikart 2008. LOSITAN: a workbench to detect molecular adaptation based on a Fst-outlier method. BMC Bioinformatics **9**:323. doi:10.1186/1471-2105-9-323.

Beaumont, M. A., and R. A. Nichols 1996. Evaluating loci for use in the genetic analysis of population structure. Proceedings of the Royal Society B: Biological Sciences **263**:1619–26. doi:10.1098/rspb.1996.0237.

Lin, F., D. S. Sepich, S. Chen, J. Topczewski, C. Yin, L. Solnica-Krezel, and H. Hamm 2005. Essential roles of G{α}12/13 signaling in distinct cell behaviors driving zebrafish convergence and extension gastrulation movements. The Journal of Cell Biology **169**:777–87. doi:10.1083/jcb.200501104.

Scholten, A., and K. W. Koch 2011. Differential calcium signaling by cone specific guanylate cyclase-activating proteins from the zebrafish retina. PloS One **6**:e23117. doi:10.1371/journal.pone.0023117.

Stein, C., M. Caccamo, G. Laird, and M. Leptin 2007. Conservation and divergence of gene families encoding components of innate immune response systems in zebrafish. Genome Biology **8**: R251. doi:10.1186/gb-2007-8-11-r251.

Tingaud-Sequeira, A., and J. Cerdà 2007. Phylogenetic relationships and gene expression pattern of three different cathepsin L (Ctsl) isoforms in zebrafish: Ctsla is the putative yolk processing enzyme. Gene **386**:98–106. doi:10.1016/j.gene.2006.08.018.

Varshney, G. K., J. Lu, D. E. Gildea, H. Huang, W. Pei, Z. Yang, S. C. Huang, et al. 2013. A large-scale zebrafish gene knockout resource for the genome-wide study of gene function. Genome Research **23**:727–35. doi:10.1101/gr.151464.112.

Weger, B. D., M. Sahinbas, G. W. Otto, P. Mracek, O. Armant, D. Dolle, K. Lahiri, et al. 2011. The light responsive transcriptome of the zebrafish: function and regulation. PloS One **6**:e17080. doi:10.1371/journal.pone.0017080.

Whiteley, A. R., A. Bhat, E. P. Martins, R. L. Mayden, M. Arunachalam, S. Uusi-Heikkilä, A. T. A. Ahmed, et al. 2011. Population genomics of wild and laboratory zebrafish (*Danio rerio*). Molecular Ecology **20**:4259–76.

**Population growth model – S7**

*Methods*

To explore the potential demographic effect of the life history evolution, we constructed a deterministic Leslie-matrix population model (Arlinghaus et al. 2009) and compared model runs with and without size-dependent harvesting. We assumed a birth-pulse population and pre-breeding census (Caswell 2001), which is appropriate for zebrafish and a common approach when modeling most fishes. We computed the population’s finite rate of increase $\lambda$ as the dominant eigenvalue of the resulting Leslie matrix $\mathbf{A}$(Caswell 2001). The form of the Leslie matrix we used was:

$\mathbf{A}=\left(\begin{matrix} f_{1}s_{0} & f_{2}s_{0} & \cdots& f_{a_{\max-1}}s_{0} & f_{a_{\max}}s_{0} \\ s_{1} & 0 & \cdots& 0 & 0 \\ 0 & s_{2} & & 0 & 0 \\ \vdots& & \ddots& \vdots& \vdots\\ 0 & 0 & \cdots& s_{a_{\max}-1} & 0 \end{matrix}\right)$, (1)

where $f_{a}$ is age-specific fecundity (egg number per female at age $a$) and $s_{a}$ is age-specific survival probability. The time unit is a day, and the maximum age was assumed $a_{\max}$= 730 (d) (i.e., 2 years).

SL at age $a$ (d) was modeled using the biphasic growth model (Lester et al. 2004) as:

$L_{a+1}=\left\{\begin{matrix} L_{a}+h & (\text{if }a<T) \\ \frac{3}{3+g}(L_{a}+h) & (\text{if }a\geq T) \end{matrix}\right.$ , (2a)

$L_{0}=ht_{1}$ , (2b)

where $g$ is reproductive investment (fraction of surplus energy invested into gonads), $h$ (mm d^-1^) is the daily juvenile growth increment and $T$ (d) is the age when fish start investing their surplus energy into reproduction. For simplicity, we modeled only females.

The age-specific fecundity $f_{a}$was defined as

$f_{a}=\left\{\begin{matrix} 0 & (\text{if }a\leq T) \\ \frac{grb_{1}L_{a}^{b_{2}}}{2w_{\text{E}}} & (\text{if }a>T) \end{matrix}\right.$ , (3)

where $w_{\text{E}}$ (mg) is the egg weight, and $r$ represents the relative caloric density of somatic tissue compared to eggs (Diana 1983). $b_{1}$and $b_{2}$ are a constant and an exponent of the allometric weight-length relationship, respectively. The number of eggs produced by a single female, that is, gonad weight divided by the egg weight, was further divided by two because only half of a particular age class was assumed to be females.

The initial age-specific survival probability $s_{a}$ until the age $a$= 10 was determined using our empirical estimates of the early life-history traits (fertilization rate, egg survival, hatching probability, and larvae survival) using data from F_9_-generation fish (see above). The age-specific survival probability $s_{a}$ ($a$>10) was determined following a published size-dependent zebrafish mortality model (Hazlerigg et al. 2012),

$s_{a}=\frac{\alpha_{a}}{1+\beta_{a}N_{a}}$, (4)

where $N_{a}$ (l^-1^) is the numerical population density of the cohort , $\alpha_{a}$ is survival at very low abundance ($N_{a}$ = 0), and $\beta_{a}$ (l) is a density-dependent mortality constant. The parameters $\alpha_{a}$ and $\beta_{a}$ were length-dependent as

$\alpha_{a}=\gamma\text{exp}(cL_{a}^{d})$ (5)

and

$\beta_{a}=\frac{p}{q}\exp(1-\frac{1}{q}L_{a})$. (6)

where $\gamma$, $c$, $d$, $p$, and $q$ are constants and an exponent of the length-mortality relationships. We could not apply the published density-dependent relationship (Hazlerigg et al. 2012) shown in equation 4 directly to our population model because the previous experiments were cohort-based (non-overlapping generations) whereas our population consisted of individuals of various ages (overlapping generations). Therefore, in all our calculations, we used the effective density (Post et al. 1999) to convert the total population effective density of the simulated population with overlapping generations to the equivalent numerical density of the cohort in a population with non-overlapping generations as

$s_{a}=\frac{\alpha_{a}}{1+\beta_{a}\sum_{i=1}^{a_{\max}} N_{i}L_{i}^{2}/L_{a}^{2}}$. (4´)

Furthermore, we made adjustments to the magnitude of the survival probability when we investigated population dynamics because the controlled experimental conditions of Hazlerigg et al. (2012) resulted in unrealistically high survival probabilities. We differentiated two life-stages (10<$a\leq$30, and 30 <$a\leq a_{\max}$) because the survival probability was reported to be low when fish were smaller than 10 mm and rather constant when fish exceeded that size (Hazlerigg et al. 2012). The value of the parameter $\gamma$ during the latter stage was determined for each selection line so that its average natural mortality during the stage equaled the instantaneous natural mortality $M$predicted from the biphasic growth model (Lester et al. 2004) for each selection line (see *Results*). Then, the survival probability during the former stage was determined by the method of Vaughan and Saila (1976) on the assumption that the population is at a specified equilibrium density. We used an effective density of 4232.5 mm^2^ l^-1^, equivalent to five individuals of random fish of the maximum size per liter, as an equilibrium population size in the absence of harvesting. Using life-history parameter values estimated for each treatment in our experiments (Table S8, see the section S2 for details of the estimation procedures), we compared the finite rate of increase $\lambda$ in each selection line. The other parameter values used in our calculations are summarized in Table S9.

We equilibrated a population that consisted of the random life-history at a certain density (an effective density of 4232.5 mm^2^ l^-1^) without fishing. Then, we calculated $\lambda$ of a variant individual from the large- or small-selected fish in the equilibrium population of random fish. The magnitude of $\lambda$ depends on the time unit used in the Leslie matrix. Therefore, we also calculated the population growth rate measured by a fixed unit of time, that is, generation time $G$ (d generation^-1^) of the random fish as $\lambda^{G}$. Generation time $G$ was calculated using the age-specific fecundity $f_{a}$ and the stable age distribution $N_{\text{s},a}$ of the population, which was obtained as an eigenvector of the Leslie matrix $\mathbf{A}$ as

$G=\frac{\sum_{i=1}^{a_{\max}} af_{i}N_{\text{s},i}}{\sum_{i=1}^{a_{\max}} f_{i}N_{\text{s},i}}$. (7)

Next, we introduced size-selective fishing mortality $F_{a}$ as

$F_{a}=\left\{\begin{matrix} F_{\max} & \text{(if } L_{1}\leq L_{a}\leq L_{2}), \\ 0 & \text{(otherwise),} \end{matrix}\right.$, (8)

where $F_{\max}$ is the maximum daily instantaneous fishing mortality, and $L_{1}$ and $L_{2}$ are the lower and upper limits of a harvest slot (mm). We considered three different types of fishing scenarios equivalent to our harvesting experiment, i.e., small-size harvested, randomly harvested, and large-size harvested fishing (Fig. S2). In the randomly harvested and large-size harvested scenarios, the upper limit of the harvest slot was set at $L_{2}=+\infty$. To mimic a 75% per-generation harvest rate of our experiments in the simulated population with overlapping generations, we determined fishing mortalities so that in random fish the equilibrium abundance of individuals of the harvested population was ~25% of the pristine population (Table S10, Fig. S2). The lower limit of the harvest slot $L_{1}$ was set at 90% of the age at maturation of random fish. The maximum instantaneous fishing mortality $F_{\max}$ of the small- and large-size harvested fishing scenarios was arbitrarily set at 200% of that of the randomly harvested fishing scenario, but we also examined the sensitivity of conclusions to alternative fishing mortality rates. When fishing was operating, the age-specific survival probabilities $s_{a}$ in the Leslie matrix (equation 1) was replaced with $s'_{a}$, which was defined as

$s'_{a}=s_{a}\exp(-F_{a})$. (9)

We calculated $\lambda$ of the variant individuals as well as the resident individuals at a fixed density because $\lambda$ varies with effective density due to the density-dependent survival probability (equation 4'). To compare the population growth rate under fishing among the treatments, we arbitrarily chose the point when the effective density declined to 20% of the pristine level because this is a common threshold of severe recruitment overfishing (Myers et al. 1994).

Uncertainty bounds of the estimated $\lambda$ were investigated by incorporating uncertainty of empirical estimates of the parameters. For each of the three main parameters (i.e., juvenile growth rate $h$, reproductive investment $g$, and maturation $T$), we sampled a new value from a normal distribution (s.d. was the empirically estimated standard error) and recalculated $\lambda$. We repeated this procedure 100 times. We also varied the maximum instantaneous fishing mortality $F_{\max}$ as well as the effective density at which $\lambda$ was calculated and evaluated the sensitivity of $\lambda$ to these parameters.

We also investigated the population dynamics of each selection line to illustrate the performance of each line with and without fishing. To that end, we imposed a certain level of fishing mortality during the first 4000 d (about 30 generations), then stopped fishing and observed the recovery processes up to the 8000 d. The starting population size was set at the equilibrium population size of the randomly selected group without fishing.

Table S8. Maturation- and growth-related parameters estimated for each treatment based on the biphasic growth model (Lester et al. 2004). Standard errors for the three main parameters are shown in parentheses.

| **Symbol** | **Treatment** | | | **Unit** |
| --- | --- | --- | --- | --- |
| *h*  *g*  *t_1_*  *T*  *M* | **Large**  0.167092  (0.006203)  0.017008  (0.000720)  -35.3146  105  (2.80)  0.014522 | **Random**  0.194131  (0.008862)  0.019956  (0.000987)  -27.9546  94  (2.99)  0.017065 | **Small**  0.189725  (0.011728)  0.020715  (0.001260)  -26.1566  91  (2.78)  0.017719 | mm  **−**  d  d  d^-1^ |

Table S9. Model parameters used in the zebrafish Leslie-matrix model.

| **Parameter** | **Symbol** | **Value** | **Unit** |
| --- | --- | --- | --- |
| Egg weight  Relative caloric density of somatic tissue to eggs  Constant in allometric weight-length relationship  Exponent of allometric weight-length relationship  Constant in length-natural mortality relationship  Exponent in length-natural mortality relationship  Constant in length-natural mortality relationship  Constant in length-natural mortality relationship | *w_E_*  *r*  *b_1_* $b_{2}$ $c$ $d$       *p*  *q* | 0.170  0.71  0.025  3.0  0.05  1.0  0.00022  6.8 | mg  −  mg mm^-^*^b^_2_*  −  mm^-d^  −  ind^-1^ mm  mm |

*Results*

Although the small-selected life-histories showed the slowest population decline among the treatments during the period when large-size harvested fishing (representing a minimum-length limit) was operating (Fig. 2F in the main text), small-selected populations showed the severest population decline of all when the other two types of fishing selectivities (unselective harvesting according to size and harvest slot) were operating (Fig. S3). This implied that life-history traits of small-selected fish were well adapted to large-size selective harvesting, but not to harvesting in general. Large-selected fish and random fish showed a similar performance when small-size harvested fishing (a harvest slot) was operating (Table 5 in the main text, Fig. S3A) but the large-selected life-history was superior to both the random-selected and the small-selected life-history in the absence of fishing.

When the maximum instantaneous fishing mortality $F_{\max}$ of the three typical fishing scenario (Fig. S2) was varied, the population growth rate $\lambda$ of large-selected life history was consistently lower than that of random-selected life history although the differences between the two were small (Fig. S4A). By contrast, the performance of small-selected life history relative to the random-selected life history varied with $F_{\max}$ as follows (Fig. S4B): when small-size harvested fishing was operating, the$\lambda$ of small-selected fish was consistently lower than that of random fish (Fig. S4B). When random-size harvested fishing was operating, the $\lambda$ of small-selected fish was also lower than that of random fish except when the fish population almost went extinct (Fig. S4B, see also Fig. S4C). However, when large-size harvested fishing was operating, the $\lambda$ of small-selected fish relative to that of random fish was higher and increased as $F_{\max}$ increased (Fig. S4B). Another important finding of the sensitivity analysis was that the population growth rate $\lambda$ of the large-selected life history relative to random life history increased as the effective density of the fish population increased (Fig. 4D). By contrast, the performance of the small-selected life history relative to the random-selected life history was largely independent of the effective density of the fish population (Fig. 4E).

Table S10. Three typical size-selective fishing scenarios.

| **Fishing scenarios** | ***F*_max_** | ***L*_1_** | ***L*_2_** |
| --- | --- | --- | --- |
| Small-size harvested  Randomly harvested  Large-size harvested | 0.018250  0.009125  0.018250 | 20.822  20.822  23.745 | 24.100  ∞  ∞ |

The lower limit of the harvest slot $L_{1}$ was set at 90% of the age at maturity of random fish. The maximum instantaneous fishing mortality $F_{\max}$ of the small- and large-size harvested scenarios is set at 200% of that of the randomly harvested scenario.

*Supporting figure legends*

Figure S2. Three typical size-selective fishing scenarios (see Table S10 for the parameter values). Red, gray, and blue symbols and lines represent large-size, random, and small-size harvested scenarios, respectively. Dashed line represents length at maturity of random fish.

Figure S3. Simulated population-level consequences of life-history changes induced by size-selective harvesting. Population recovery was monitored after (A) small-size harvested (a variant of a harvest slot) or (B) randomly harvested fishing operated for the first 4000 days. Red, gray, and blue symbols and lines represent populations of small-selected, random, and large-selected fish, respectively.

Figure S4. Sensitivity of the population growth rate $\lambda$. (A – C) $F_{\max}$ of the three fishing scenarios (Fig. S2, Table S10) were varied. (A) $\lambda$ of large-selected fish relative to random fish. (B) $\lambda$ of small-selected fish relative to the random fish. (C) Equilibrium density of random fish relative to the pristine population (i.e., $F_{\max}$ = 0). $F_{\max}$of the three typical fishing scenarios were determined to mimic a 75% per-generation harvest rate of our experiments (i.e. the relative equilibrium density is 0.25, dotted line). (D, and E) $\lambda$ under the three fishing scenarios was calculated at various effective density relative to the pristine population (i.e., $F_{\max}$ = 0). (D) $\lambda$ of large-selected fish relative to random fish. (E) $\lambda$ of small-selected fish relative to the random fish. Red, gray, and blue symbols and lines represent large-size, random, and small-size harvested scenarios, respectively.

*Literature*

Arlinghaus, R., S. Matsumura, and U. Dieckmann 2009. Quantifying selection differentials caused by recreational fishing: development of modeling framework and application to reproductive investment in pike (*Esox lucius*). Evolutionary Applications **2**:335–55. doi:10.1111/j.1752-4571.2009.00081.x.

Caswell, H. 2001. Matrix Population Models. Sunderland. Sinauer Associates.

Diana, J. S. 1983. Growth, maturation, and production of Northern pike in three Michigan lakes. Transactions of the American Fisheries Society **112**:38–46.

Hazlerigg, C. R. E., K. Lorenzen, P. Thorbek, J. R. Wheeler, and C. R. Tyler 2012. Density-dependent processes in the life history of fishes: evidence from laboratory populations of zebrafish *Danio rerio*. PloS One **7**:e37550. doi:10.1371/journal.pone.0037550.

Lester, N. P., B. J. Shuter, and P. A. Abrams 2004. Interpreting the von Bertalanffy model of somatic growth in fishes: the cost of reproduction. Proceedings of the Royal Society B: Biological Sciences **271**:1625–31. doi:10.1098/rspb.2004.2778.

Meyers, R. A., A. A. Rosenberg, P. M. Mace, N. Barrowman, and V. R. Restrepo 1994. In search of thresholds for recruitment overfishing. ICES Journal of Marine Science **51**:191-205.

Post, J. R., E. A. Parkinson, and N. T. Johnston 1999. Density-dependent processes in structured fish populations: interaction strenghts in whole-lake experiments. Ecological Monographs **69**:155–75.

Vaughan, D. S., and S. B. Saila 1976. A method for determining mortality rates using the Leslie matrix. Transactions of the American Fisheries Society **105**:380–83.
